# A disubstituted aniline probe for enhanced peroxidase-based proximal protein labelling

**DOI:** 10.1039/d5cb00095e

**Published:** 2025-10-08

**Authors:** Pornchai Kaewsapsak, Nattavorapon Tantisasirat, Sucheewin Krobthong, Peeraphan Compiro, Ariya Khamwut, Kidakarn Ratchakitprakarn, Naphat Chantaravisoot, Kriangsak Faikhruea, Withsakorn Sangsuwan, Medena Noikham, Worawan Bhanthumnavin, Tirayut Vilaivan, Sunchai Payungporn, Yodying Yingchutrakul, Watthanachai Jumpathong, Chanat Aonbangkhen

**Affiliations:** a Department of Biochemistry, Faculty of Medicine, Chulalongkorn University, Pathum Wan Bangkok 10330 Thailand; b Center of Excellence in System Microbiology (CESM), Faculty of Medicine, Chulalongkorn University, Pathum Wan Bangkok 10330 Thailand; c Center of Excellence in Natural Products Chemistry (CENP), Department of Chemistry, Faculty of Science, Chulalongkorn University, Pathum Wan Bangkok 10330 Thailand chanat.a@chula.ac.th; d Organic Synthesis Research Unit (OSRU), Department of Chemistry, Faculty of Science, Chulalongkorn University, Pathum Wan Bangkok 10330 Thailand; e National Center for Genetic Engineering and Biotechnology, NSTDA Pathum Thani 12120 Thailand; f Program on Chemical Sciences, Chulabhorn Graduate Institute, Lak Si Bangkok 10210 Thailand; g Chulabhorn Royal Academy Bangkok 10210 Thailand

## Abstract

Proteins are biomolecules essential for cellular functions, including cell signaling and regulation. Protein misfolding or mislocalisation can result in various diseases. Peroxidase-mediated proximity labelling has emerged as a powerful tool for studying subcellular proteome and protein–protein interactions. However, the traditional probe, biotin-phenol, suffers from limitations including low protein enrichment efficiency, and the formation of oxidised and polymerised products, complicating the downstream analysis. To address these challenges, a novel probe, *N*-(4-amino-3,5-dimethylbenzyl)desthiobiotinamide (DBA-Me), for protein labelling in living cells was developed. Western blot analysis demonstrated efficient labelling of bovine serum albumin *in vitro*. Liquid chromatography-tandem mass spectrometry (LC-MS/MS) data confirmed the formation of one-to-one adducts from the *in vitro* labelling reaction. Notably, this novel probe (DBA-Me) also exhibited labelling activity towards nucleic acids. Moreover, DBA-Me also permits APEX2-mediated labelling within the mitochondrial matrix of HEK293FT cells, and demonstrated improved recovery of labelled proteins after streptavidin enrichment compared to the conventional biotin-phenol (BP) probe, highlighting its superior potential application *in cellulo*. This facilitates peroxidase-mediated proximity labelling applications in subcellular localisation of proteins, and protein structures, with broader implications for understanding cellular processes and disease mechanisms.

## Abstract 2

Scheme of peroxidase-catalyzed proximity-based labeling by a novel homo-disubstituted aniline biotin probe.

## Graphical abstract

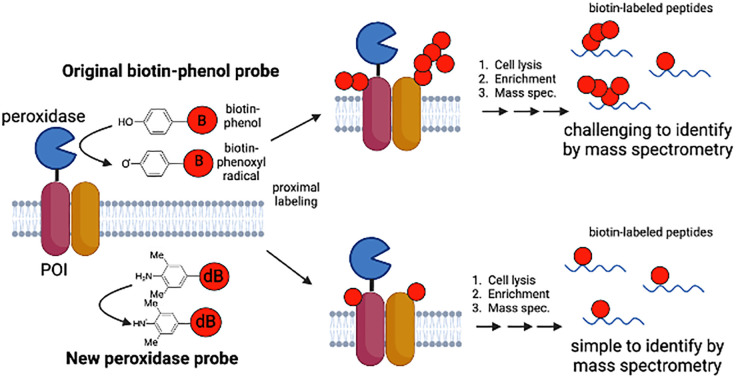

## Introduction

Proteins constitute the molecular workhorses of cellular function, orchestrating a myriad of biological processes essential for life. From enzymatic catalysis to structural support, proteins play pivotal roles in virtually every aspect of cellular physiology.^1–3^ Central to their functionality is their precise spatial organisation within subcellular compartments, a phenomenon known as subcellular localisation. The spatial distribution of proteins governs their interactions with other cellular components, dictating cellular signalling pathways,^4^ metabolic fluxes,^5^ and structural integrity.^6^ Moreover, the intricate network of protein–protein interactions underpins the dynamic regulation of cellular processes. Disruptions in these interactions, whether through mislocalisation or aberrant binding partners, can perturb cellular homeostasis and contribute to disease pathogenesis.^4,7–11^

Recently enzyme-catalysed proximity-based labelling techniques have emerged as transformative tools for dissecting the intricacies of subcellular dynamics. These methodologies harness genetically engineered enzymes, strategically fused with targeting motifs, to orchestrate the precise labelling of proximal biomolecules. Notably, BioID exploits the biotin ligase BirA to enzymatically activate biotin,^12^ facilitating its covalent attachment to nearby proteins at lysine residues.^13^ However, BioID exhibits diminished activity below physiological temperatures,^14^ limiting its utility in certain contexts. In addition, the endogenous biotin level could give rise to background biotinylation. In contrast, the PUP-IT approach leverages the PafA enzyme to tag Pup protein onto proximal proteins,^15^ albeit with a large protein tag and prolonged labelling times unsuitable for investigations of rapid turnover interactions. Alternatively, peroxidase-based techniques by horseradish peroxidase (HRP) and engineered ascorbate peroxidase (APEX) catalyse the conversion of biotin-phenol (BP) to biotin-phenoxyl radicals upon H_2_O_2_ addition,^16^ enabling the selective labelling of electron-rich amino acid residues like tyrosine. While HRP has higher activity, its performance is compromised in reducing environments due to disulfide bonds.^17,18^ In contrast, APEX exhibits versatility across both reducing and oxidising conditions but has lower activity. Hence, an A134P mutation was introduced into APEX, resulting in APEX2 with high activity.^19^ Beyond protein labelling, APEX has also been employed to elucidate protein–nucleic acid interactions, extending its utility to the interrogation of DNA and RNA dynamics.^20^ Thus, the advent of enzyme-catalysed proximity labelling techniques not only augments our understanding of protein dynamics but also heralds novel avenues for probing biomolecular interactions with unprecedented precision and depth.

**Fig. 1:**
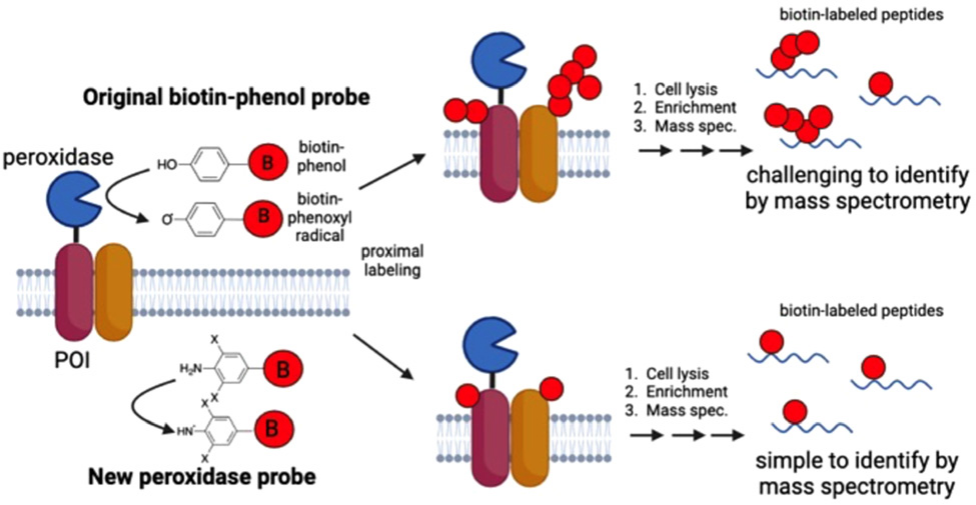
Scheme of peroxidase-catalysed proximity-based labelling by the novel homo-disubstituted aniline biotin probe.

Although biotin-phenol (BP) has been used as a substrate for peroxidase in many studies, it suffers from several drawbacks. Lee *et al.* observed that sample enrichment *via* streptavidin magnetic beads results in low recovery rates due to the strong binding affinity between biotin and streptavidin.^21^ Additionally, the sulphur atom in biotin can be oxidised, generating more diverse adducts. To address these, they introduced desthiobiotin-phenol (DBP), which not only enhances recovery but also yields stronger mass spectrometry signals.^21^ While BP has been extensively used in proteomics, its reactivity towards nucleic acids is low. Ying Zhou *et al.* expanded its utility by introducing biotin-aniline (BA), which exhibits improved reactivity towards nucleic acids while retaining protein labelling activity.^22^ Lastly, the presence of a vacant *ortho* position in the aromatic ring of BP makes it susceptible to polymerisation,^23,24^ complicating omics analysis and data interpretation. Here, novel diortho-substituted biotin-aniline substrates were tested to address these aforementioned challenges in peroxidase-mediated proximity labelling (Fig. 1).

## Results and discussion

### Screening of diortho-substituted biotin-aniline probes

**Fig. 2:**
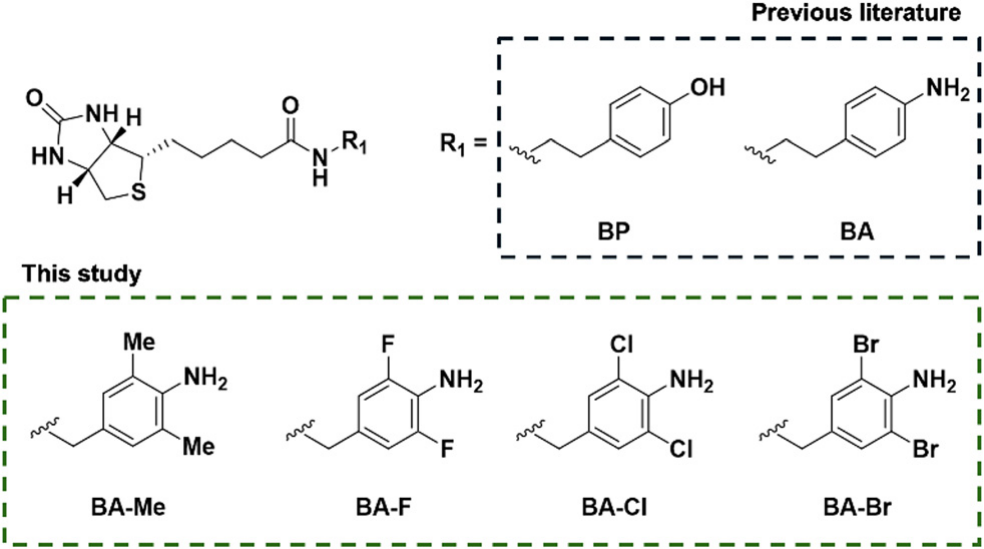
Diortho-substituted biotin-aniline probes in this study.

**Fig. 3:**
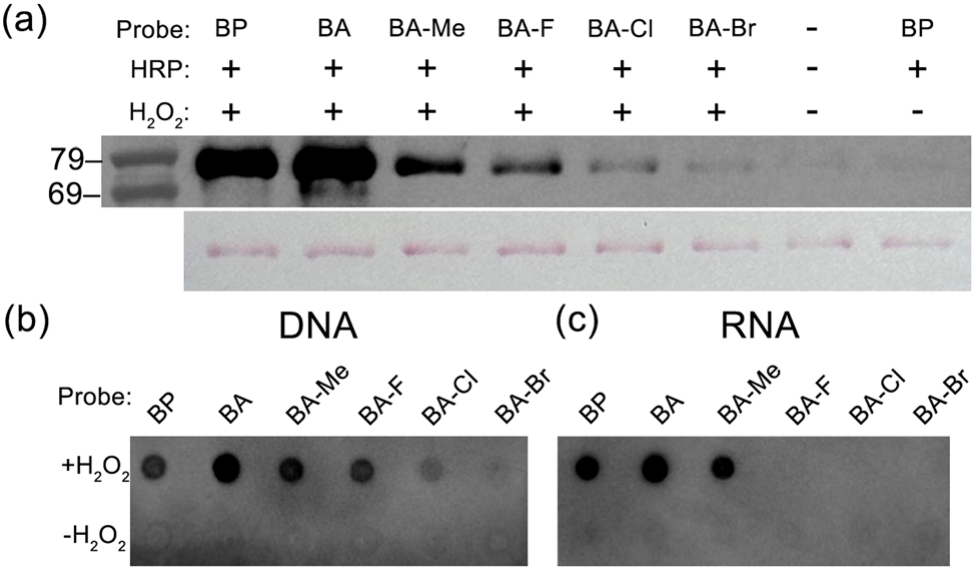
Screening probe reactivity on proteins and nucleic acids *via* a horseradish peroxidase-mediated labelling reaction. (a) Protein labelling with bovine serum albumin (BSA) followed by SDS-PAGE and western blot analysis. Top: SA-HRP blotting; bottom: Ponceau S staining. (b) Dot-blot image of single strand DNA labelling. (c) Dot-blot image of yeast RNA labelling.

To prevent polymerisation of the probe together with expanding applications in nucleic acids research, homo-diortho-substituted biotin-aniline derivatives (BA-Me, BA-F, BA-Cl, and BA-Br) were synthesised by coupling the corresponding benzylamine derivatives to biotin (Fig. 2). Firstly, these probes were tested for reactivity with bovine serum albumin (BSA) as a model protein to assure the labelling activity on proteins with horseradish peroxidase by western blot. Biotinylated proteins were probed by streptavidin-conjugated horseradish peroxidase (SA-HRP). The chemiluminescence signal was given by all of the probes. Among the new probes, the signal was strongest with BA-Me, followed by BA-F and BA-Cl, and weakest with BA-Br (Fig. 3a). According to the mechanism of oxidation of aniline by peroxidase, aniline is oxidised with dissociation of the N–H bond to form an anilino radical *via* redox reaction.^25^ Hence, the high reactivity of BA-Me is possibly due to the electronic effect of the electron-donating methyl group, which lowers the bond dissociation energy of the N–H bond^26^ and *E*_ox_^27^ making BA-Me more prone to oxidation by peroxidase compared to its counterparts with electron-withdrawing halogen groups. To assess labelling efficiency toward nucleic acids, single-stranded DNA and yeast RNA were used as DNA and RNA models, respectively, in the presence of HRP. In a dot blot assay using single-stranded DNA, the labelling trend mirrored that observed in protein labelling: BA-Me showed the highest signal, followed by BA-F and BA-Cl, and a very faint signal was observed with BA-Br (Fig. 3b). Interestingly, in the RNA labelling assay, only BA-Me yielded a detectable signal (Fig. 3c). This suggests that BA-Me is the only derivative among the four capable of labelling RNA effectively. The limited reactivity of BA-F, BA-Cl, and BA-Br with RNA may be attributed to the inherent structural complexity and fragility of yeast-derived RNA compared to single-stranded DNA.

**Fig. 4:**
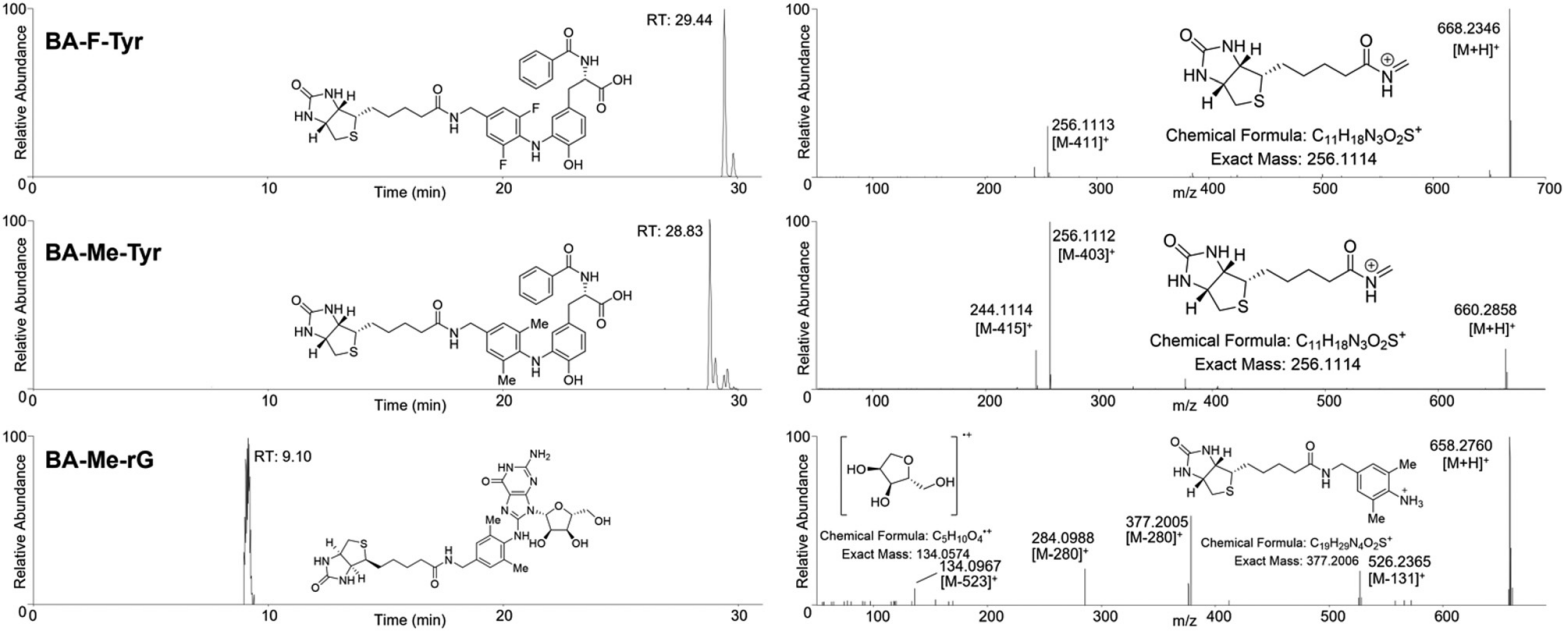
LC-MS/MS analysis for horseradish peroxidase-mediated labelling reactions with *N*-benzoyl tyrosine and guanosine. (Top) Adduct of BA-Me with *N*-benzoyl tyrosine. (Middle) Adduct of BA-F with *N*-benzoyl tyrosine. (Bottom) Adduct of BA-Me with guanosine. For each row of adducts, (left) chromatogram of selected mass, and (right) MS2 spectra of each selected peak from the chromatogram.

To confirm the formation of one-to-one adducts from labelling, the reaction products were analysed and verified using LC-MS/MS. The reaction between *N*-benzoyl tyrosine, a representative for proteins residues, and the probes in the presence of horseradish peroxidase (HRP) revealed that the new probes (BA-Me and BA-F) as well as BP and BA formed an adduct with *N*-benzoyl tyrosine ([M + H]^+^: 660.2847, 668.2346, 647.2532, and 646.2692, respectively) (Fig. 4 and Fig. S1–S4) and also their oxidised forms. In contrast, no adduct formation with BA-Cl and BA-Br was detected (Fig. S5 and S6). Furthermore, only BA-Me together with BP and BA formed an adduct with guanosine ([M + H]^+^: 658.2761, 645.2448, and 644.2673, respectively) (Fig. 4) along with their oxidised adducts (Fig. S7–S12). This is the first time that the labelled tyrosine and guanosine were confirmed by LC-MS/MS. However, further LC-MS/MS experiments with other nucleotides (rA, rC, rU, and dT) did not find detectable adducts with BP and BA-Me (Fig. S13 and S14). These results corresponded to the previous results from western blot and dot blot. Therefore, BA-Me was determined as the best candidate for further experiments.

### Characterising the reactivity of a novel desthiobiotin probe *in vitro*

**Fig. 5:**
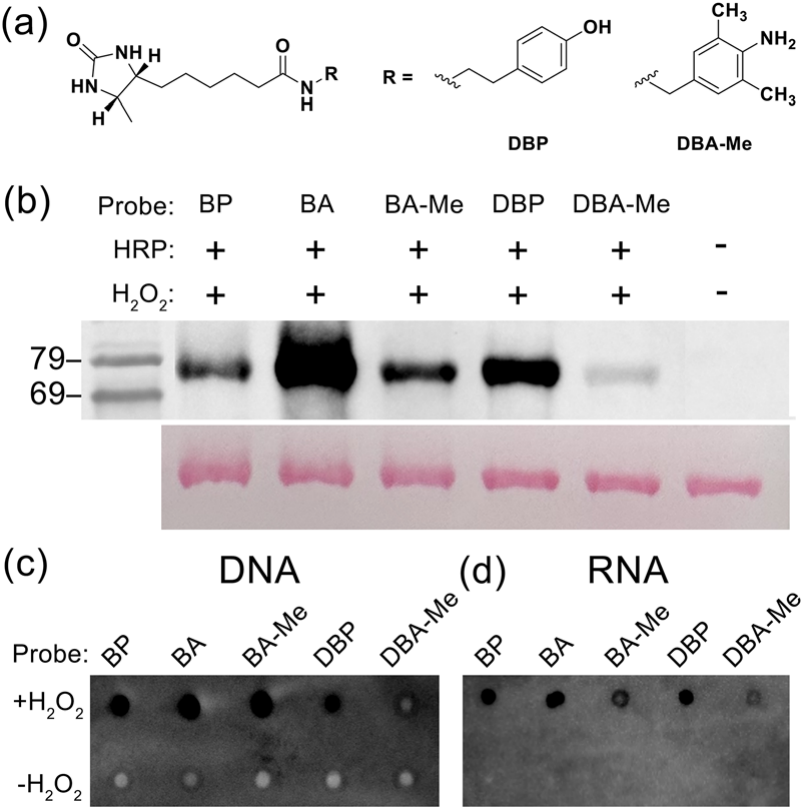
DBA-Me probe reactivity on proteins and nucleic acids *via* horseradish peroxidase-mediated labelling reaction. (a) Structure of the desthiobiotin probes. (b) Protein labelling with bovine serum albumin (BSA) followed by SDS-PAGE and western blot analysis. (c) Dot-blot image of single strand DNA labelling. (d) Dot-blot image of yeast RNA labelling.

According to the screening results, BA-Me was selected for further experiments. To minimise the formation of oxidised adducts associated with the sulfide group in biotin, 4-amino-3,5-dimethylbenzylamine was conjugated to desthiobiotin instead of biotin, thereby preventing potential oxidation reactions. To verify the reactivity of the novel probe, *N*-(4-amino-3,5-dimethylbenzyl)desthiobiotinamide (DBA-Me) was tested by western blot and dot-blot assay. The results showed that DBA-Me retained labelling activity toward proteins, DNA, and RNA. However, the obtained signal was reduced possibly due to lower binding affinity to streptavidin of desthiobiotin compared to biotin, which may amplify the apparent difference in biotinylation level (Fig. 5).

### Identification of labelled peptides by LC-MS/MS

**Fig. 6:**
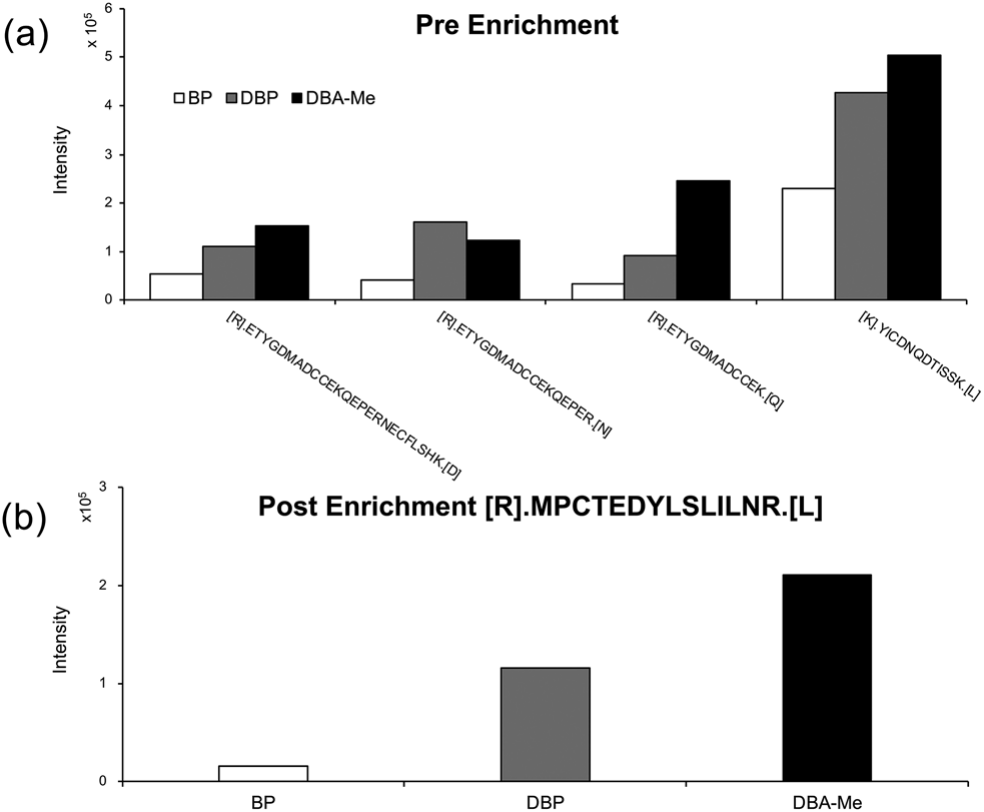
Mass intensity detected by LC-MS/MS of the labelled peptides. (a) Pre enrichment and (b) Post enrichment by streptavidin magnetic beads.

The presence of sulphur in BP contributes to the formation of oxidised labelled peptides, thereby reducing the intensity of peptide signals detected by mass spectrometry. Additionally, the exceptionally strong affinity between biotin and streptavidin hinders the efficient recovery of labelled peptides during enrichment using streptavidin-conjugated magnetic beads. Furthermore, the unsubstituted *ortho* position on the benzene ring is prone to undergo polymerisation, resulting in heterogeneous products that complicate interpretation by LC-MS/MS. To circumvent these issues, BSA was labelled with each probe (BP, DBP, and DBA-Me), followed by LC-MS/MS analysis. The chromatograms of pre enrichment peptides from the three probes showed a similar profile suggesting that all samples were uniformly digested (Fig. S15–S17). Prior to enrichment, peptides labelled with desthiobiotin-based probes showed higher mass intensity than those labelled with BP, with DBA-Me yielding the highest signal in three out of four labelled peptides: ([R].ETYGDmADCCEKQEPERNECFLSHK.[D], [R].ETYGDMADCCEK.[Q], and [K].YICDNQDTISSK.[L]) (Fig. 6). This trend of increasing mass intensity from BP to DBP and DBA-Me was also observed after streptavidin-based enrichment. The mass intensity of a distinct peptide, [R].MPCTEDYLSLILNR.[L], labelled by DBA-Me was nearly 14 fold higher than that of the conventional probe, BP, and approximately 1.8 fold higher than that of DBP. These results suggest that the improved signal from DBA-Me-labelled peptides reflects greater product homogeneity, making DBA-Me the most effective probe among those tested.

### Labelling of mitochondrial matrix proteins by DBA-Me in HEK293FT cells

**Fig. 7:**
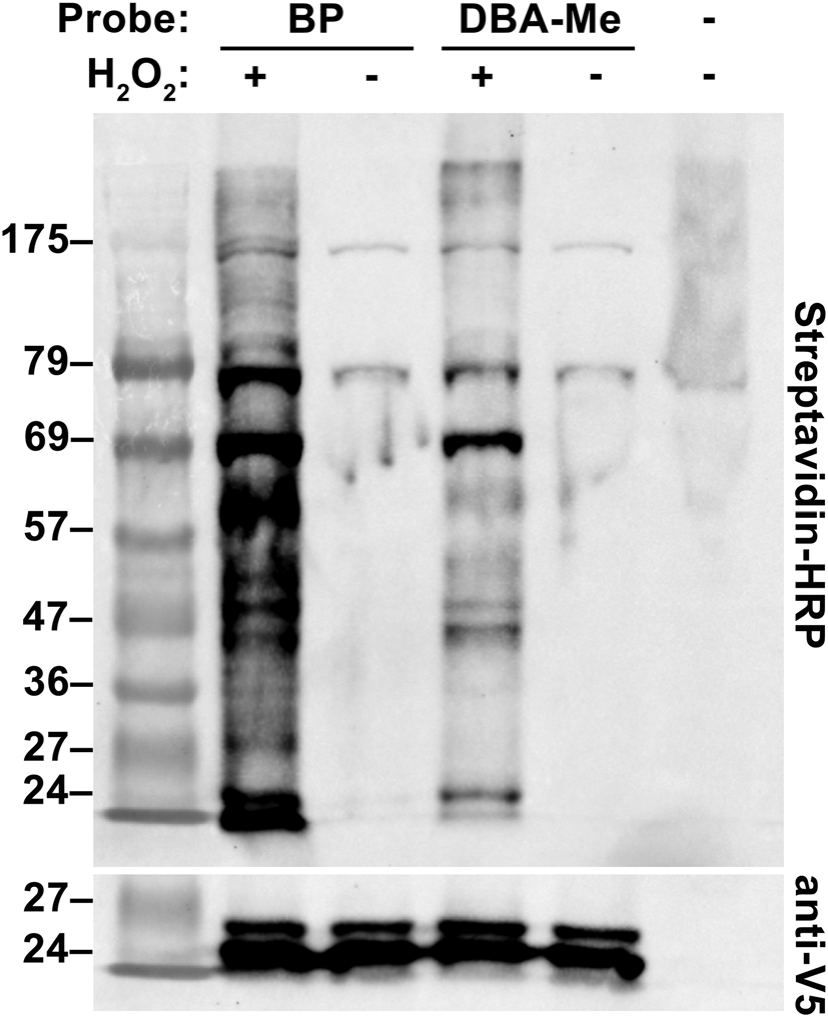
Western blot analysis of APEX2-mediated mitochondrial matrix protein labelling in HEK293FT. (Top) Streptavidin-HRP (bottom) anti-V5.

**Fig. 8:**
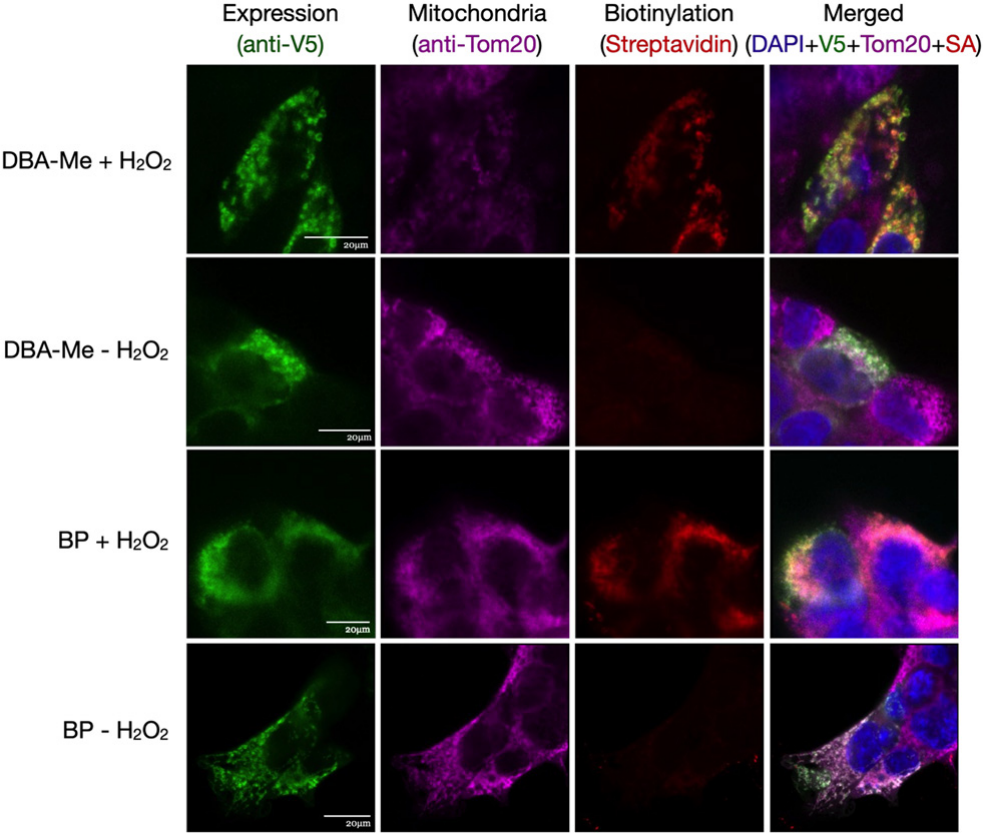
Immunofluorescence of APEX2-mediated biotinylation *in cellulo*. HEK293FT cells expressing V5-tagged mito-APEX2 were biotinylated, fixed, and stained as indicated. Rows 1 and 3 show cells treated with DBA-Me and BP in the presence of H_2_O_2_, while rows 2 and 4 display the same conditions but without H_2_O_2_ treatment. Scale bars, 20 μm. SA = Streptavidin.

To ensure suitability for live-cell applications, the cytotoxicity of the probes was first evaluated using MTT assay, and no significant cytotoxic effects were observed in HEK293FT cells (Fig. S18). HEK293FT cells expressing mitochondrial matrix-targeted APEX2 (mito-V5-APEX2) were incubated with DBA-Me and treated with H_2_O_2_ to evaluate the cell permeability and live-cell labelling. Western blot analysis confirmed biotinylation of intracellular proteins (Fig. 7), indicating that DBA-Me can cross the cell membrane and label endogenous targets. To further assess its spatial specificity, HEK293FT cells expressing mito-V5-APEX2 were labelled and analysed by immunofluorescence imaging. As shown in Fig. 8, biotinylated proteins were specifically localised to the mitochondrial matrix under both DBA-Me and BP labelling conditions. Additionally, immunofluorescence imaging of DBA-Me and BP in cells expressing LMNA-APEX2 showed comparable biotinylation in the nucleoplasm, implying similar labelling radius (Fig. S19). These findings demonstrate that DBA-Me not only functions effectively in live cells but also achieves labelling comparable to the conventional BP probe, highlighting its potential as a novel and robust tool for proximity-based labelling in live-cell applications.

### Enrichment of labelled proteins in HEK293FT cells

**Fig. 9:**
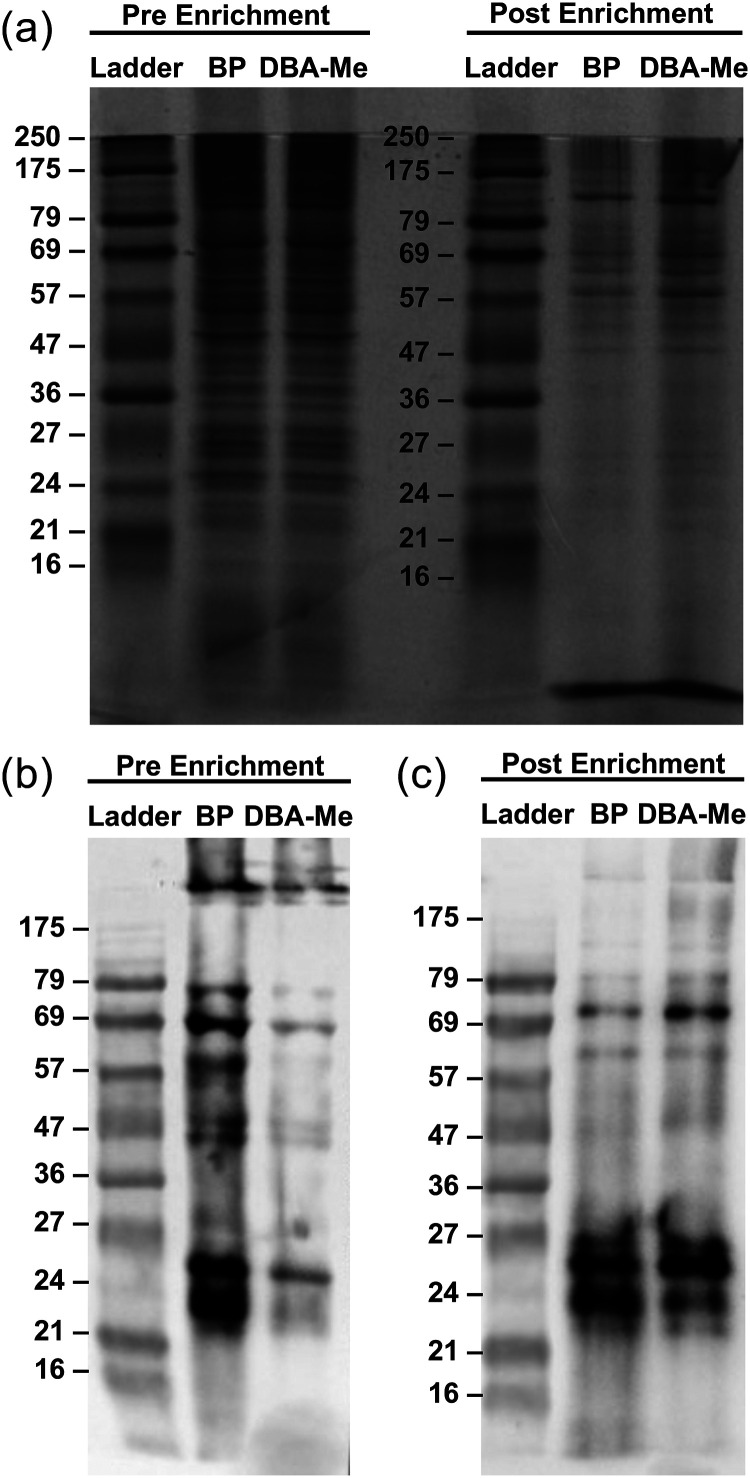
Evaluation of protein recovery from streptavidin enrichment using APEX2-mediated mitochondrial protein labelling in HEK293FT cells. (a) Coomassie-stained SDS-PAGE gel. Lanes: 1, 4 = protein ladder; 2 = pre enrichment (BP); 3 = pre enrichment (DBA-Me); 5 = post enrichment (BP); 6 = post enrichment (DBA-Me). (b) SA-HRP staining blot of pre enrichment. Lane 1: protein ladder, lane 2: BP, lane 3: DBA-Me. (c) SA-HRP staining blot of post enrichment. Lane 1: protein ladder, lane 2: BP, lane 3: DBA-Me.

To evaluate whether the DBA-Me allows better recovery of biotinylated proteins in a cellular context than the traditional biotin-phenol (BP) probe, HEK293FT cells expressing mito-V5-APEX2 were treated with either BP or DBA-Me. The cell lysates were then enriched by streptavidin magnetic beads and the recovered proteins were determined by Coomassie staining. Strikingly, despite the lower SA-HRP signal in the initial lysates (Fig. S38), the post-enrichment from DBA-Me-labelled cells recovered more biotinylated proteins than BP as the intensity of the proteins bands for DBA-Me is higher across different molecular weights (Fig. 9). This suggested that although DBA-Me showed less biotinylation signal in the pre-enrichment stage, probably due to less polymerized adducts, it did recover more biotinylated proteins. Taken together, these results demonstrate that while DBA-Me may produce less SA-HRP signal in total lysate blots, its key advantage lies in its dramatically improved elution efficiency. The ability to recover a larger quantity and broader range of labeled proteins makes DBA-Me a more effective and practical probe for preparing samples for downstream proteomic analysis.

## Conclusions

The use of BP, the conventional probe used in peroxidase-mediated proximity labelling, is limited by low recovery efficiency during streptavidin-based enrichment and the formation of oxidised or polymerised byproducts. In this study, DBA-Me was developed as a novel peroxidase probe that addresses these limitations and demonstrates strong potential for proximity-based labelling applications. Crucially, in a direct comparison using APEX2-mediated labelling in HEK293FT cells, DBA-Me demonstrated improvement in the recovery of labelled proteins after streptavidin enrichment compared to conventional BP. This superior elution efficiency, confirmed by both Coomassie blue staining and western blotting, represents a practical advantage for downstream proteomic applications, directly overcoming one of the key bottlenecks in the workflow.

Furthermore, LC-MS/MS analysis confirmed the formation of a defined one-to-one adduct between DBA-Me and tyrosine, allowing for more accurate interpretation of proteomics data, including studies of protein topology, structure, and interactomes. Successful labelling of mitochondrial matrix proteins *via* APEX2-mediated proximity labelling established its compatibility with live-cell applications, such as confocal imaging and proteomics. Additionally, the formation of a one-to-one adduct with guanosine suggests the potential of DBA-Me for nucleic acid labelling, broadening its applicability to spatial genomics and transcriptomics through the subcellular mapping of both proteins and nucleic acids.

## Author contributions

Conceptualisation, project administration, supervision: P. K., C. A., W. J.; resources, funding acquisition: P. K., C. A., W. J., T. V., S. P., Y. Y.; investigation, methodology: P. K., C. A., W. J., T. V.; Data curation: N. T., S. K., K. R., K. F., P. C., A. K., N. C., W. S.; formal analysis: N. T., S. K., P. C., A. K., M. N, W. S., C. A., P. K.; validation: N. T., W. S., C. A., P. K., W. J., T. V., W. B., writing – original draft: N. T., P. K., C. A., writing – review & editing: inputs from all authors.

## Conflicts of interest

There are no conflicts to declare.

## Supplementary Material

CB-006-D5CB00095E-s001

CB-006-D5CB00095E-s002

## Data Availability

The data supporting this article have been included as part of the supplementary information (SI). Supplementary information is available. See DOI: https://doi.org/10.1039/d5cb00095e.
