# Supplementary material for: A disubstituted aniline probe for enhanced peroxidase-based proximal protein labelling
Source: RSC Chem Biol. 2025 Oct 8;6(12):1861–6. doi: 10.1039/d5cb00095e (PMC12571195; doi:10.1039/d5cb00095e)
Supplement: CB-006-D5CB00095E-s001 [file CB-006-D5CB00095E-s001.pdf]

Supplementary Information (SI) for RSC Chemical Biology.
This journal is © The Royal Society of Chemistry 2025

Supporting Information

A disubstituted aniline probe for enhanced peroxidase-based proximal protein labelling

Pornchai Kaewsapsak[a,b,†], Nattavorapon Tantisasirat[c,†], Sucheewin Krobthong[c], Peeraphan Compiro[a,b], Ariya Khamwut[b], Kidakarn Ratchakitprakarn[b], Naphat Chantaravisoot[a,b], Kriangsak Faikhruea[d], Withsakorn Sangsuwan[c], Medena Noikham[d], Worawan Bhanthumnavin[d], Tirayut Vilaivan[d], Sunchai Payungporn[a,b], Yodying Yingchutrakul[e], Watthanachai Jumpathong[f,g], and Chanat Aonbangkhen[c,*]

[a] Department of Biochemistry, Faculty of Medicine, Chulalongkorn University, Pathum Wan, Bangkok 10330, Thailand

[b] Center of Excellence in System Microbiology (CESM), Faculty of Medicine, Chulalongkorn University, Pathum Wan, Bangkok 10330, Thailand

[c] Center of Excellence in Natural Products Chemistry (CENP), Department of Chemistry, Faculty of Science, Chulalongkorn University, Pathum Wan, Bangkok 10330, Thailand

[d] Organic Synthesis Research Unit (OSRU), Department of Chemistry, Faculty of Science, Chulalongkorn University, Pathum Wan, Bangkok 10330, Thailand

[e] National Center for Genetic Engineering and Biotechnology, NSTDA, Pathum Thani, 12120, Thailand

[f] Program on Chemical Sciences, Chulabhorn Graduate Institute, Lak Si, Bangkok 10210, Thailand

[g] Chulabhorn Royal Academy, Bangkok, 10210, Thailand

[*]Corresponding Author, E-mail: chanat.a@chula.ac.th and watthanachai@cgi.ac.th

Table of Contents

EXPERIMENTAL PROCEDURE **3**

SUPPORTING FIGURES AND TABLES **13**

FIGURE S1. 13
FIGURE S2. 14
FIGURE S3. 15
FIGURE S4. 16
FIGURE S5. 17
FIGURE S6. 18
FIGURE S7. 19
FIGURE S8. 20
FIGURE S9. 21
FIGURE S10. 22
FIGURE S11. 23
FIGURE S12. 24
FIGURE S13. 25
FIGURE S14. 26
FIGURE S15. 27
FIGURE S16. 28
FIGURE S17. 29
FIGURE S18. 30
FIGURE S19. 30

NMR SPECTRA **31**

FIGURE S20. 31
FIGURE S21. 32
FIGURE S22. 33
FIGURE S23. 34
FIGURE S24. 35
FIGURE S25. 36
FIGURE S26. 37
FIGURE S27. 38
FIGURE S28. 39
FIGURE S29. 40
FIGURE S30. 41
FIGURE S31. 42

RAW IMAGE DATA **43**

FIGURE S32. 43
FIGURE S33. 44
FIGURE S34. 45
FIGURE S35. 46
FIGURE S36. 46
FIGURE S37. 47
FIGURE S38. 48

Experimental Procedure

Chemical Abbreviations

$BH_3 \cdot SMe_2$	Borane dimethylsulfide
CuCN	Copper cyanide
DCM	Dichloromethane
DIPEA	Diisopropylethylamine
DMF	*N,N*-dimethylformamide
ECL	Enhanced chemiluminescence
FBS	Fetal bovine serum
H_2O_2	Hydrogen peroxide
HATU	Hexafluorophosphate azabenzotriazole tetrametyl uronium
HRP	Horseradish peroxidase
KBr	Potassium bromide
$KBrO_3$	Potassium bromate
$LiAlH_4$	Lithium aluminum hydride
NMP	*N*-methyl-2-pyrrolidone
SDS	Sodium dodecyl sulfate
SDS-PAGE	Sodium dodecyl sulfate-Polyacrylamide gel electrophoresis
THF	Tetrahydrofuran

General Information

Unless otherwise mentioned, all chemicals and reagents were purchased from Sigma-Aldrich, Alfa Aesar, or TCI and used directly without further purification. All solvents were degassed. Thin layer chromatography (TLC) analyses were performed on TLC Silica gel 60 F_{254} (Sigma-Aldrich) and visualized by fluorescence quenching under UV light and by staining ninhydrin, or potassium permanganate, respectively. Flash column chromatography was performed on Silica Gel 60 (300–400 Mesh. Proton nuclear magnetic resonance spectroscopy (^{1}H NMR) and Carbon nuclear magnetic resonance spectroscopy (^{13}C NMR) spectra were recorded on a FT-NMR spectrometer (JNM-ECZR series) (500 MHz) and processed by MestReNova software. The NMR spectra were shown in Figure S16-27. The chemical shifts (δ) were reported in parts per million (ppm). Coupling constants (J) were given in Hertz (Hz), signal shapes and splitting patterns are indicated as follows: s, singlet; d, doublet;

t, triplet; m, multiplet; dd, doublet of doublet; ddd, doublet of doublet of doublet. High-resolution mass spectra were obtained by Q Extractive Focus mass spectrometer (Thermo Scientific) equipped with an electrospray ion source was also used. Avantor® Hichrom C18 column (5 µm, 250 mm x 4.6 mm id).

Synthesis of Peroxidase Probes

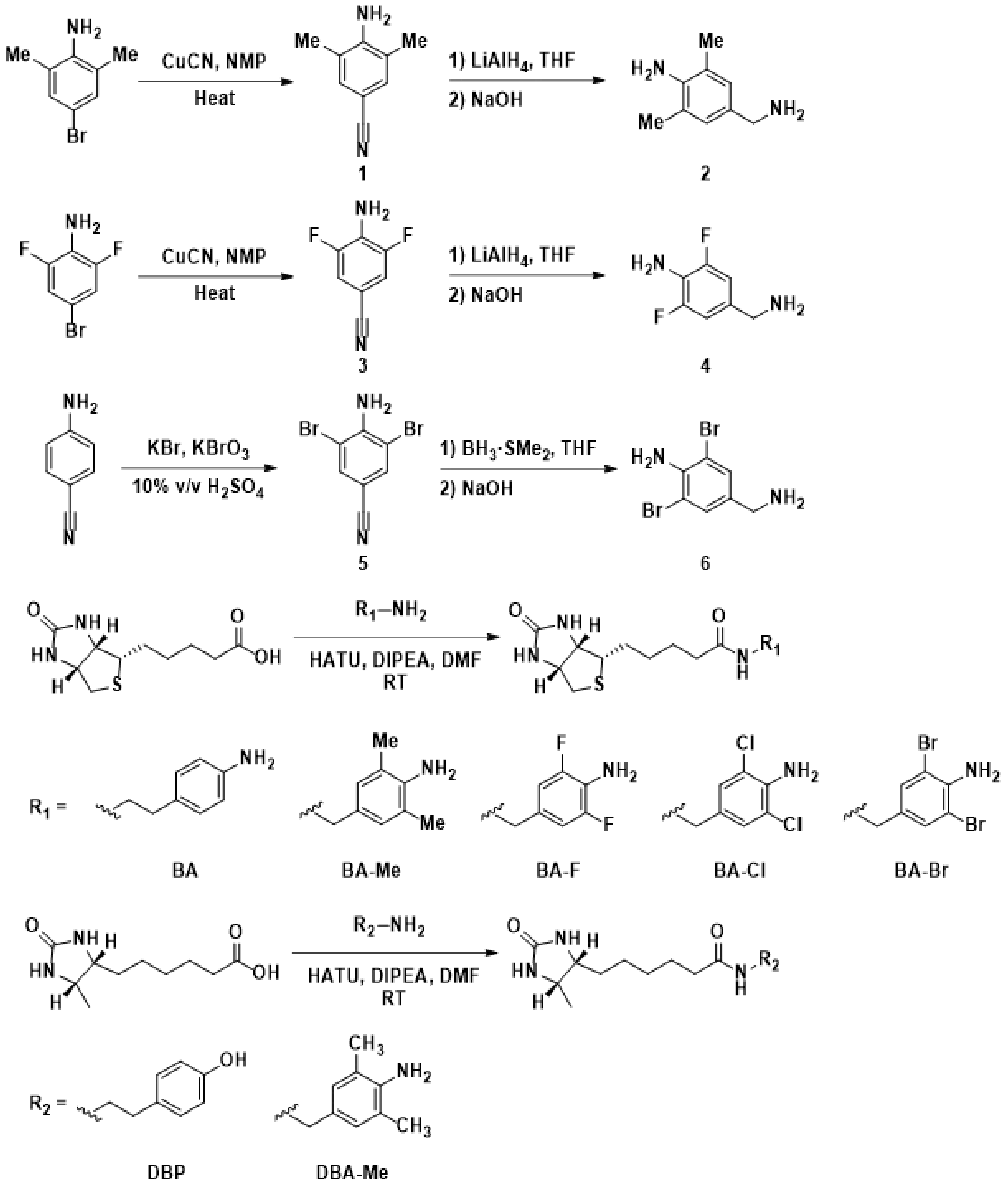

4-Amino-3,5-dimethylbenzonitrile (1). The solution of 4-bromo-2,6-dimethylaniline (500 mg, 2.50 mmol, 1.0 eq) and copper cyanide (448 mg, 5.00 mmol, 2.0 eq) in anhydrous NMP (5 mL) was stirred at 160 ºC for 12 hours. After completion, the reaction mixture was cooled down to room temperature and ethylenediamine (0.67 mL, 10.0 mmol, 4.0 eq) was added. The mixture was extracted by ethyl acetate and water. The combined organic layers were washed with 10% v/v ethylenediamine followed by water and dried in vacuo. The crude was purified by silica gel flash column chromatography with 20% ethyl acetate in hexane as mobile phase to obtain the pure product as a pale yellow solid (303.0 mg, 83%). ^{1}H NMR (500 MHz, Chloroform-*d*) δ 7.20 (s, 2H), 4.07 (s, 2H), 2.16 (s, 6H). The spectroscopic data is consistent with the previous report.[1]

4-Amino-3,5-dimethylbenzylamine (2). To a cooled (-20 ºC) suspension of $LiAlH_4$ (208 mg, 5.47 mmol, 4.0 eq) in anhydrous THF under inert atmosphere was added dropwise the solution of 4-amino-3,5-dimethylbenzonitrile (200 mg, 1.37 mmol, 1.0 eq) in anhydrous THF (10 mL). The reaction mixture was warmed to the room temperature and then refluxed for 5 hours. After cooling down to 0 ºC, diethyl ether was added followed by water and 15% w/v sodium hydroxide to deactivate excess $LiAlH_4$. The mixture was filtered through celite and the filtrated was concentrated under vacuo to get the crude product as a pale yellow oil, which was used in the next step without further purification.

4-Amino-3,5-difluorobenzonitrile (3). As described in the synthesis of **1**, 4-bromo-2,6-difluoroaniline (500 mg, 2.40 mmol, 1.0 eq) was reacted with copper cyanide (431 mg, 4.81 mmol, 2.0 eq) in anhydrous NMP (5 mL). The crude product was purified by silica gel flash column chromatography with 20% ethyl acetate in hexane as mobile phase to obtain pure product as a pale orange pink solid (221 mg, 60%). ^{1}H NMR (500 MHz, Chloroform-*d*) δ 7.14 (dd, J = 6.1, 2.2 Hz, 2H), 4.29 (s, 2H). The spectroscopic data is consistent with the previous report.[2]

4-Amino-3,5-difluorobenzylamine (4). As described in the synthesis of **2**, the reduction of 4-amino-3,5-difluorobenzonitrile (133 mg, 0.86 mmol, 1.0 eq) by $LiAlH_4$ (131 mg, 3.45 mmol, 4.0 eq) in anhydrous THF (10 mL) was performed to give the crude product as a colorless oil, which was used in the next step without further purification.

4-Amino-3,5-dichlorobenzylamine. The compound was obtained from Tokyo Chemical Industry CO., LTD.

4-Amino-3,5-dibromobenzonitrile (5). To a suspension of 4-aminobenzonitrile (200 mg, 1.69 mmol) in 10% v/v sulfuric acid (2 mL) at room temperature was added dropwise the solution of potassium bromide (268 mg, 2.25 mmol, 1.33 eq) and potassium bromate (187 mg, 1.12 mmol, 0.66 eq) in water (2.5 mL). The reaction mixture was stirred for 3 hours. The precipitate was collected by filtration, washed with water, and dried in oven at 100 ºC for 30 min. After purification with silica gel flash column chromatography using 20% ethyl acetate in hexane as the mobile phase, the product was obtained as a white to pale pink solid (821 mg, 88%). ^{1}H NMR (500 MHz, Chloroform-*d*) δ 7.66 (s, 2H), 5.11 (s, 2H). ^{13}C NMR (126 MHz, Chloroform-*d*) δ 146.14, 117.36, 107.62, 101.98. The spectroscopic data is consistent with the previous report.[3]

4-Amino-3,5-dibromobenzylamine (6). To a cooled (0 ºC) solution of 4-amino-3,5-dibromobenzonitrile (138 mg, 0.50 mmol, 1 eq) in anhydrous THF 5 mL) was added $BH_3 \cdot SMe_2$ (0.1 mL, 1.00 mmol, 2 eq) dropwise. The reaction mixture was refluxed overnight and then cooled down to room temperature. Water was slowly added until no effervescence followed by concentrated HCl solution in equal volume of water. The resulting mixture was refluxed for 1 hour. After removal of the solvent under vacuo, the dried reaction mixture was reconstituted by DCM, basified with 15% NaOH solution, and extracted with DCM several times. The organic layers were combined, dried by anhydrous sodium sulfate, and concentrated under vacuo. The crude product was purified by silica gel column chromatography using 1:9:190 (25% NH_3:MeOH:DCM) as the mobile phase to obtain the pure compound as a white solid (112 mg, 80%). ^{1}H NMR (500 MHz, DMSO-*d6*) δ 7.38 (s, 2H), 5.14 (s, 2H), 3.53 (s, 2H), 3.34 (s, 2H). ^{13}C NMR (126 MHz, Chloroform-*d*) δ 141.04, 135.20, 130.68, 107.67, 44.13.

General procedure for biotinylation of benzylamine derivatives

To a cooled (0 ºC) solution of biotin (122 mg, 0.50 mmol, 1.0 eq), HATU (285 mg, 0.75 mmol, 1.5 eq), and DIPEA (194 mg, 1.50 mmol, 3 eq) in anhydrous DMF (3 mL) was added the solution of corresponding primary amine (0.50 mmol, 1 eq) in DMF (2 mL) dropwise. The reaction mixture was stirred overnight at room temperature. The solvent was removed in vacuo and the crude product was purified by silica gel flash column chromatography with the gradient solvent system of 2% to 10% methanol in dichloromethane. The pure product was obtained as a white solid.

***N*-(4-aminophenethyl)biotinamide (BA)** ^{1}H NMR (500 MHz, DMSO-d_6) δ 7.81 (t, J = 5.7 Hz, 1H), 6.83 (d, J = 8.3 Hz, 2H), 6.48 (d, J = 8.4 Hz, 2H), 6.45 (s, 1H), 6.38 (s, 1H), 4.85 (s, 2H), 4.33 – 4.29 (m, 1H), 4.14 – 4.10 (m, 1H), 3.17 – 3.12 (m, 2H), 3.10 – 3.06 (m, 1H), 2.82 (dd, J = 12.5, 5.1 Hz, 1H), 2.57 (d, J = 12.4 Hz, 1H), 2.50 (t, J = 7.5 Hz, 2H), 2.03 (t, J = 7.4 Hz, 2H), 1.64 – 1.39 (m, 4H), 1.34 – 1.20 (m, 2H). ^{13}C NMR (126 MHz, DMSO-d_6) δ 172.05, 162.91, 146.83, 129.09, 126.47, 114.08, 61.15, 59.31, 55.56, 40.73, 39.97, 35.33, 34.62, 28.30, 28.14, 25.44. HRMS (ESI) m/z: $[M+H]^+$ Calcd for $C_{18}H_{27}N_4O_2S$ 363.1849; Found 363.1840. The spectroscopic data is consistent with the previous report.[4]

***N*-(4-amino-3,5-dimethylbenzyl)biotinamide (BA-Me)**: ^{1}H NMR (500 MHz, DMSO-d_6) δ 8.04 (t, J = 5.7 Hz, 1H), 6.68 (s, 2H), 6.45 (s, 1H), 6.37 (s, 1H), 4.43 (s, 2H), 4.30 (dd, J = 7.8, 5.0 Hz, 1H), 4.16 – 4.08 (m, 1H), 4.03 (d, J = 5.8 Hz, 2H), 3.11 – 3.04 (m, 1H), 2.81 (dd, J = 12.4, 5.1 Hz, 1H), 2.57 (d, J = 12.4 Hz, 1H), 2.08 (t, J = 7.4 Hz, 2H), 2.05 (s, 6H), 1.66 – 1.40 (m, 4H), 1.38 – 1.22 (m, 2H). ^{13}C NMR (126 MHz, DMSO-d_6) δ 172.18, 163.27, 143.52, 127.75, 126.89, 120.92, 61.57, 59.71, 56.02, 42.37, 40.41, 35.68, 28.76, 28.56, 25.89, 18.39. HRMS (ESI) m/z: $[M+H]^+$ Calcd for $C_{19}H_{29}N_4O_2S$ 377.2006; Found 377.2009.

***N*-(4-amino-3,5-difluorobenzyl)biotinamide (BA-F)**: ^{1}H NMR (500 MHz, DMSO-*d6*) δ 8.22 (t, J = 5.9 Hz, 1H), 6.77 (dd, J = 7.6, 2.1 Hz, 2H), 6.43 (s, 2H), 6.36 (s, 1H), 5.07 (s, 4H), 4.32 – 4.28 (m, 1H), 4.13 – 4.11 (m, 1H), 4.10 (d, J = 6.0 Hz, 7H), 3.08 (ddd, J = 8.6, 6.1, 4.4 Hz, 1H), 2.82 (dd, J = 12.5, 5.1 Hz, 1H), 2.57 (d, J = 12.3 Hz, 1H), 2.11 (t, J = 7.4 Hz, 6H), 1.66 – 1.41 (m, 4H), 1.38 – 1.23 (m, 2H). ^{13}C NMR (126 MHz, DMSO-d_6) δ 172.63, 163.28, 151.45 (dd, J = 238.6, 9.3 Hz), 127.16 (t, J = 7.4 Hz), 124.45 (t, J = 16.9 Hz), 110.42 (dd, J = 15.4, 6.3 Hz), 61.56, 59.71, 55.97, 41.48, 40.40, 35.64, 28.76, 28.54, 25.81. HRMS (ESI) m/z: $[M+H]^+$ Calcd for $C_{17}H_{23}F_2N_4O_2S$ 385.1504; Found 385.1505.

***N*-(4-amino-3,5-dichlorobenzyl)biotinamide (BA-Cl)**: ^{1}H NMR (500 MHz, DMSO-d_6) δ 8.25 (t, J = 6.0 Hz, 1H), 7.10 (s, 2H), 6.45 (s, 1H), 6.37 (s, 1H), 5.41 (s, 2H), 4.30 (dd, J = 7.8, 4.9 Hz, 1H), 4.11 (ddd, J = 7.8, 4.4, 1.7 Hz, 1H), 4.08 (d, J = 5.8 Hz, 2H), 3.11 – 3.03 (m, 1H), 2.81 (dd, J = 12.4, 5.1 Hz, 1H), 2.57 (d, J = 12.4 Hz, 1H), 2.10 (t, J = 7.4 Hz, 2H), 1.66 – 1.40 (m, 4H), 1.37 – 1.21 (m, 2H). ^{13}C NMR (126 MHz, DMSO-d_6) δ 172.10, 162.76, 139.87, 128.73, 127.06, 117.86, 61.05, 59.20, 55.50, 40.73, 39.91, 35.14, 28.22, 28.04, 25.32. HRMS (ESI) m/z: $[M+H]^+$ Calcd for $C_{17}H_{23}Cl_2N_4O_2S$ 417.0913; Found 417.0902.

***N*-(4-amino-3,5-dibromobenzyl)biotinamide (BA-Br)**: ^{1}H NMR (500 MHz, DMSO-*d6*) δ 8.21 (t, J = 6.0 Hz, 1H), 7.26 (s, 2H), 6.39 (s, 1H), 6.32 (s, 1H), 5.21 (s, 2H), 4.32 – 4.22 (m, 1H), 4.10 – 4.06 (m, 1H), 4.04 (d, J = 5.9 Hz, 2H), 3.03 (ddd, J = 8.5, 6.3, 4.4 Hz, 1H), 2.78 (dd, J = 12.5, 5.1 Hz, 1H), 2.53 (d, J = 12.4 Hz, 1H), 2.06 (t, J = 7.4 Hz, 2H), 1.62 – 1.37 (m, 4H), 1.33 – 1.19 (m, 2H). ^{13}C NMR (126 MHz, DMSO-d_6) δ 172.08, 162.74, 141.55, 130.91, 130.30, 107.45, 61.04, 59.20, 55.48, 40.48, 39.91, 35.13, 28.19, 28.03, 25.31. HRMS (ESI) m/z: $[M+H]^+$ Calcd for $C_{17}H_{23}Br_2N_4O_2S$ 504.9903; Found 504.9907.

***N*-(4-hydroxylphenethyl)desthiobiotinamide (DBP):** ^{1}H NMR (500 MHz, Methanol-d_4) δ 7.02 (d, J = 8.5 Hz, 2H), 6.70 (d, J = 8.4 Hz, 2H), 3.86 – 3.76 (m, 1H), 3.72 – 3.66 (m, 1H), 3.35 (t, J = 7.3 Hz, 2H), 2.69 (t, J = 7.2 Hz, 2H), 2.15 (t, J = 7.4 Hz, 2H), 1.62 – 1.54 (m, 2H), 1.50 – 1.21 (m, 6H), 1.11 (d, J = 6.4 Hz, 3H). HRMS (ESI) m/z: $[M+H]^+$ Calcd for $C_{18}H_{28}N_3O_3$ 334.2125; Found 334.2119. The spectroscopic data is consistent with the previous report.[5]

***N*-(4-amino-3,5-dimethylbenzyl)biotinamide (DBA-Me)**: ^{1}H NMR (500 MHz, DMSO-*d6*) δ 8.03 (t, J = 5.8 Hz, 1H), 6.68 (s, 2H), 6.32 (s, 1H), 6.13 (s, 1H), 4.44 (s, 2H), 4.03 (d, J = 5.8 Hz, 2H), 3.64 – 3.55 (m, 1H), 3.50 – 3.44 (m, 1H), 2.07 (t, J = 7.5 Hz, 2H), 2.05 (s, 6H), 1.54 – 1.44 (m, 2H), 1.38 – 1.13 (m, 6H), 0.95 (d, J = 7.7 Hz, 3H). ^{13}C NMR (126 MHz, DMSO-d_6) δ 172.20, 163.36, 143.52, 127.70, 126.90, 120.88, 55.49, 50.73, 42.32, 35.81, 30.10, 29.28, 26.15, 25.81, 18.41, 16.03. HRMS (ESI) m/z: $[M+H]^+$ Calcd for $C_{19}H_{31}N_4O_2$ 347.2442; Found 347.2440.

Evaluation of probe reactivity by mass spectrometry. Either 100 μM guanosine representing DNA/RNA labelling or 1 mM *N*-benzoyl-tyrosine representing protein labelling was labelled with 500 μM probes and 2.25 μM horseradish peroxidase in PBS. Reaction was initiated by adding 1 mM H_2O_2. After 1 min of reaction time, reaction was terminated by 50% v/v methanol. The solution was analyzed by UPLC-MS

The UPLC separation in the UPLC-MS system was operated under DIONEX Ultimate 3000 Rapid Separation (RS) at a flow rate of 200 μL/min over 30 min with gradient ranging from 95% solvent A (0.1% formic acid in water) to 70% solvent B (acetonitrile). The mass spectrometer used in the system is Q Exactive Focus mass spectrometer (Thermo Scientific) equipped with an electrospray ion source was also used. Avantor® Hichrom C18 column (5 μm, 250 mm x 4.6 mm) was used to separate the mixture. Analytical column was placed in a column oven at the controlled temperature of 30 ºC.

To assess the reactions of BA-Me with other nucleosides, 100 μM of each nucleoside (adenosine, cytosine, uridine, and thymidine) was labelled with BA-Me using the same mobile phases elution and the same UPLC-MS instrumental system as described above for guanosine labelling except that the mixture was separated by Cogent UDC Cholesterol column (4 um, 250 x 3 mm) under the controlled temperature of 30 ºC. The gradient elution was set up by ramping from 5%B to 75%B within 40 min at the flow rate of 300 μL/min.

Evaluation of probe reactivity on RNA/DNA by dot blot analysis. Either 2 μM polyG 64 mer for DNA or 250 μg/mL yeast RNA for RNA was labelled with 500 μM probes and 2.25 μM horseradish peroxidase in PBS at room temperature. Reaction was initiated by adding 1 mM H_2O_2. After 1 min of reaction time, reaction was terminated by adding quencher solution. The labelled DNA and RNA were purified by viral RNA extraction kit (Zymo Research) with using 10 μL DEPC water as the final eluent. After labelling and purification had been done, 1 μL of purified DNA or RNA was dotted onto Hybond®-N+ hybridization membranes and crosslinked by UV irradiation with 2500 x100 μJ (Stratalinker). The membrane was incubated in 1:3000 Streptavidin-HRP in 0.1% Tween20 PBST for

15 min. The membrane was washed with 0.1% Tween20 PBST for 3x5 min followed by washing with PBS for 5 min respectively. The membrane was developed in ECL (Merck) and imaged on ChemiDoc Imaging System (Bio-Rad).

Evaluation of probe reactivity on protein by western blot analysis. To label protein, 5 μg of BSA was labelled with 500 μM probes and 2.25 μM horseradish peroxidase in PBS at room temperature. Reaction was initiated by 1 mM H_2O_2. After 1 min of reaction time, reaction was terminated by adding quencher solution. Proteins were denatured by 1x Leammli buffer and incubated at 95 ºC for 5 min. Proteins were separated by 10% SDS-PAGE gel. Then, proteins will be transferred to nitrocellulose membrane with Trans-Blot Turbo Transfer System (Bio-Rad) under 1.3A 25V for 20 min. After blot transfer, the membrane was blocked by 5% skimmed milk in TBST (0.1% Tween20) for 1 hour. Then, the membrane will be incubated in 1:5000 Streptavidin-HRP in PBST for 1 hour. The membrane was washed with PBST for 3x5 min followed by washing with PBS for 5 min respectively. The membrane was developed in ECL (Merck) and imaged on ChemiDoc Imaging System (Bio-Rad).

Analysis of labelled sites from bovine serum albumin (BSA) by high resolution tandem mass spectrometry (LC-MS/MS/MS). To label protein, 500 μg of BSA was labelled with 500 μM probes and 2.25 μM HRP in PBS at room temperature. Reaction was initiated by 1 mM H_2O_2. After 30 minute of reaction time, reaction was terminated by adding quencher solution (final concentration of 10 mM sodium ascorbate and 10 mM of sodium azide). The labelled BSA was subsequently enriched using streptavidin-conjugated magnetic beads. Protein purification was performed via acetone precipitation at a volumetric ratio of 1:5 (sample:acetone) and incubated at –20°C for 16 hours. The resulting protein pellet was collected and reconstituted in 0.15% RapiGest SF dissolved in 10 mM ammonium bicarbonate. Carbamidomethylation, enzymatic digestion using trypsin, and sample desalting were performed following previously established protocols without modification[6]. Quantification of the resulting peptide concentration was conducted using the Pierce™ Quantitative Peptide Assay Kit. The tryptic peptides were then subjected to LC-MS/MS/MS analysis, based on a previously reported method with minor adjustments[7]. Spectral acquisition was performed in positive ion mode using an Orbitrap HF Hybrid Quadrupole-Orbitrap™ mass spectrometer coupled to a nano-LC system. Chromatographic separation was achieved with a Thermo Scientific™ ES902 C18 reverse-phase column (75 μm × 25 cm, 2.0 μm particle size), employing a linear gradient from 7% to 50% of mobile phase B over 115 minutes at a flow rate of 300 nL/min. The mobile phases consisted of 0.1% formic

acid in water (mobile phase A) and 95% acetonitrile with 0.1% formic acid (mobile phase B). Peptide detection was carried out using a data-dependent acquisition (DDA) strategy combined with higher-energy collisional dissociation (HCD). Full MS scans were recorded over a mass-to-charge (*m/z*) range of 420–1600, with an automatic gain control (AGC) target of 3×10^6 ions and a resolution of 120,000. MS/MS scans were triggered based on the same AGC target and acquired at a resolution of 30,000, with normalized collision energy set to 32. Post-acquisition, the identification of modified tyrosine residues was performed using Proteome Discoverer software. The analysis utilized the UniProtKB reference sequence for bovine albumin (P02769-ALBU_BOVIN) for peptide-spectrum matching and site localization. The total ion chromatograms obtained from the LC-MS/MS/MS runs were examined to assess the efficiency of enzymatic digestion, as indicated by the distribution and intensity of peptide ion signals across the chromatographic profile.

Mammalian cell culturing and probe labelling. HEK 293FT cells were obtained and cultured in a 24-well cell culture plate (SPL Life Sciences) supplemented by DMEM (Gibco) supplemented with 10% FBS, 50 units/mL penicillin, and 50 μg/mL streptomycin at 37 ºC under 5% CO_2. To induce the protein expression in cells, cells at 60-70% confluency were used. Lipofectamine 2000 (Life Technologies), typically with 60 μL Lipofectamine 2000 and 12,000 ng of the APEX2 plasmid per 6-well plate. Cells were incubated for 3 hours, then media was changed to fresh DMEM with 10% FBS, 50 units/mL penicillin, and 50 μg/mL streptomycin. After overnight, Cell media was changed to 500 μM of each probe in MEM+ and incubated at 37 °C under 5% CO_2 for 30 min. After that, cells were labelled in 4 conditions: 1. **DBA-Me**, 2. **DBA-Me** with 1 mM H_2O_2 for 1 min, 3. **BP**, and 4. **BP** with 1 mM H_2O_2 for 1 min. The reaction was then quenched by replacing with a quenching solution (PBS containing 10 mM sodium azide and 10 mM sodium ascorbate). Then, the cells were washed again with the quenching solution. Cells were detached using cold quenching solution and centrifuged at 2000 × g for 10 min at 4 °C. The cell pellets were flash frozen in liquid nitrogen for storage.

For western blotting, the cell pellets were lysed with RIPA lysis buffer (50 mM Tris, 150 mM NaCl, 0.1% SDS, 0.5% sodium deoxycholate, 1% Triton X-100) with 1x protease cocktail (Sigma Aldrich, P8849), for 5 min at 0 °C. Lysates were clarified by centrifugation at 16,000 × g for 10 min at 4 ºC. Then, the cell lysates were probed with Streptavidin-HRP similar to western blotting of BSA as mentioned earlier.

Cytotoxicity assay. Cytotoxicity was evaluated using the MTT assay based on an in-house protocol. HEK293FT cells were seeded in 96-well plates at a density of 1×10^4 cells/mL and incubated for 24 hours at 37 °C in 5% CO_2. Cells were then treated with desthiobiotin probes (BP, BA, BA-Me, DBA-Me) at concentrations of 250, 500, and 750 µM and incubated for an additional 24 hours. After treatment, the culture media were removed, and cells were incubated with MTT solution (3-(4,5-dimethylthiazol-2-yl)-2,5-diphenyltetrazolium bromide; Sigma-Aldrich, Cat. No. M5655) at 5 mg/mL in PBS for 3 hours at 37 °C. The resulting formazan crystals were dissolved in DMSO, and absorbance was measured at 570 nm using a PerkinElmer EnSight Multimode Microplate Reader to determine cell viability.

Cell lysate enrichment by streptavidin magnetic beads. The protein concentrations of the labelled cell lysates were measured by BCA assay (Merck) and then adjusted the concentration to 5000 µg/mL. 30 µL of streptavidin magnetic beads (Merck) was washed with PBS once and added to 240 µL of the sample. The suspension was rotated for an hour at room temperature. The beads were washed by PBS three times and eluted by 30 µL of 95% formamide 10 mM EDTA (pH 8.0) at 95 ºC for 10 min. To precipitate the proteins, 500 µL of cold acetone was added to the eluate and incubated at -20 ºC overnight. The sample was centrifuged 16,000 × g for 10 min at 4 ºC. The protein pellets were reconstituted using 30 µL of RIPA buffer and the protein concentration was quantified by BCA assay. Post enrichment samples were denatured by 1x Laemmli buffer and incubated at 95 ºC for 5 min. Both of pre and post enrichment sample were separated by 10% SDS-PAGE gel and stained by Imperial™ Protein Stain (Thermo Scientific).

Immunofluorescence. The labelled cells were fixed with 4% paraformaldehyde (PFA) in PBS at 0°C for 10 minutes, followed by two washes with PBS. Permeabilization was performed using cold methanol at -20 °C for 5 minutes, and cells were subsequently washed three times with PBS. To detect APEX2-fusion protein expression, cells were incubated at 4°C for 1.5 hours with mouse anti-V5 antibody (Invitrogen, cat. no. R960-25, 1:600 dilution in 1% BSA/PBS) and/or rabbit anti-Tom20 antibody (1:300 dilution in 1% BSA/PBS). After three PBS washes, cells were incubated at room temperature for 1 hour with secondary antibodies: Alexa Fluor 488-goat anti-rabbit IgG (Invitrogen, cat. no. A-11001, 1:1000 dilution in 1% BSA/PBS), Texas Red-goat anti-mouse IgG (1:400 dilution in 1% BSA/PBS), and Alexa Fluor 647 Streptavidin (BioLegend, cat. no. 405237, 1:1000 dilution in 1% BSA/PBS). DAPI (3.33 µg/mL in 1% BSA/PBS) was added and incubated for 10 minutes. Cells

were then washed three times with PBS on ice. Imaging was performed using Zeiss LSM800 Airyscan confocal microscope (Zeiss, Jena, Germany).

Supporting Figures and Tables

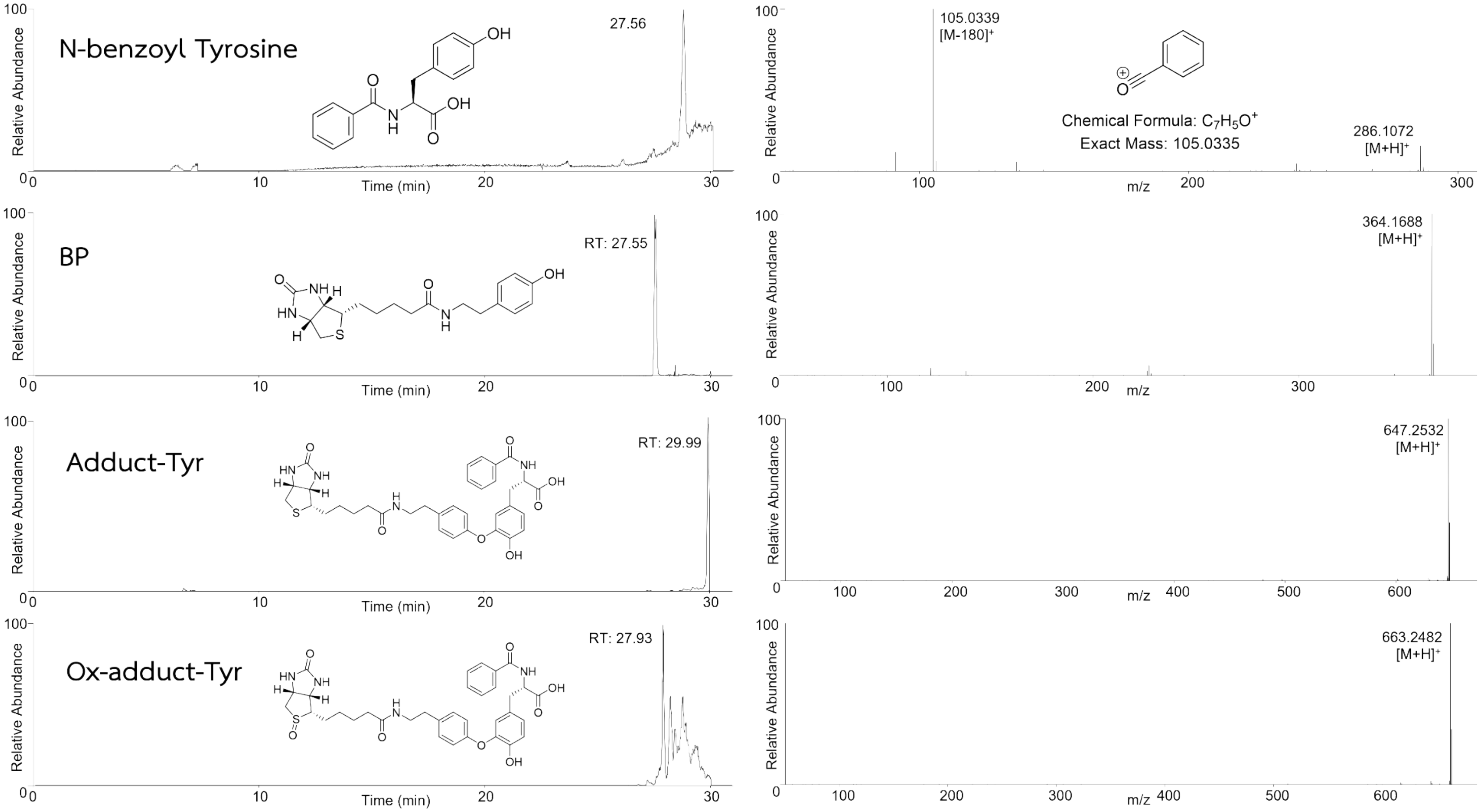

Figure S1. LC-MS/MS results of one-by-one adduct identification from horseradish peroxidase-mediated labeling reaction between **BP** and *N*-benzoyl tyrosine. For each row of adduct, (Left) Chromatogram, (Right) Mass spectra of each selected peak of chromatogram by targeted mass.

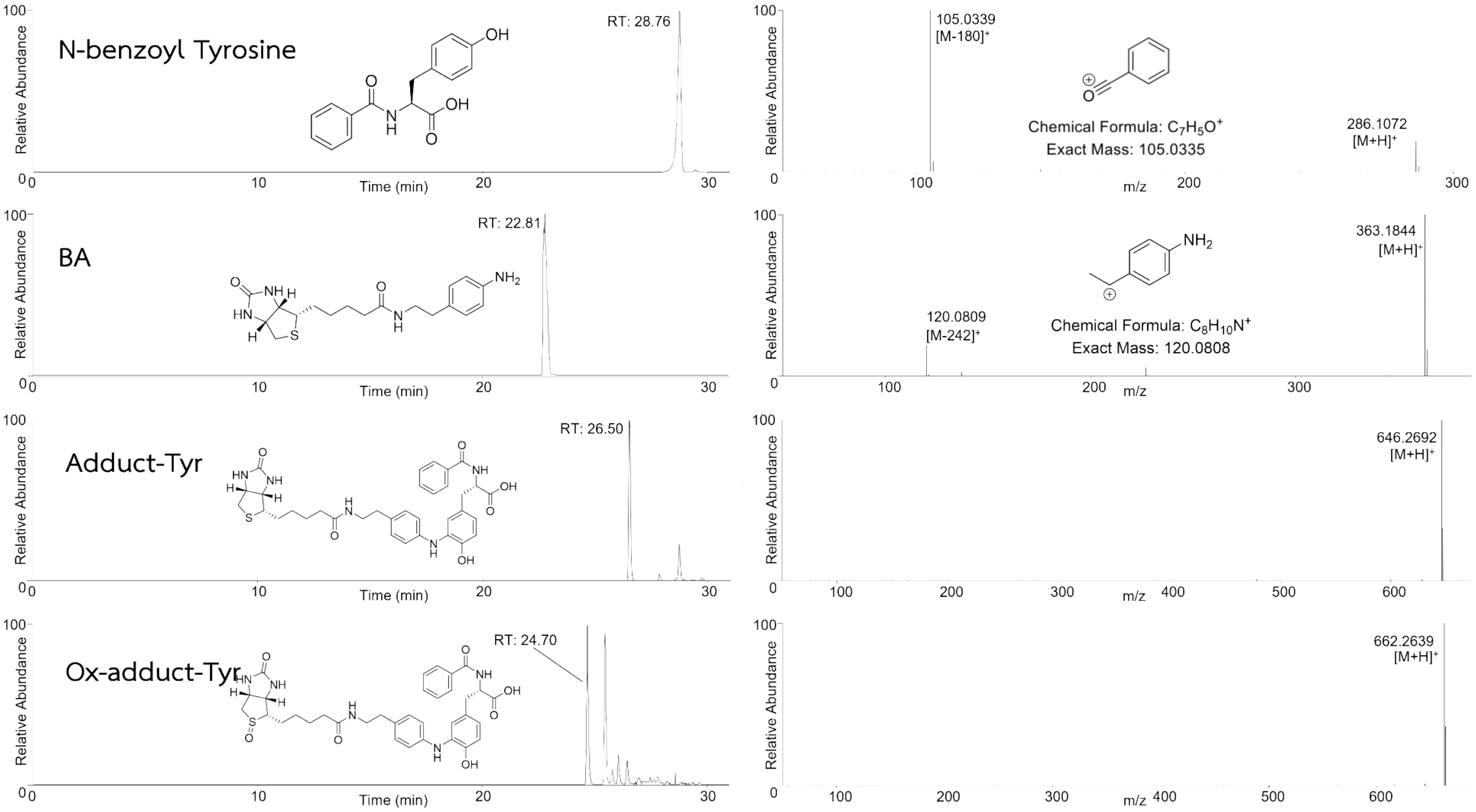

Figure S2. LC-MS/MS results of one-by-one adduct identification from horseradish peroxidase-mediated labeling reaction between **BA** and *N*-benzoyl tyrosine. For each row of adduct, (Left) Chromatogram, (Right) Mass spectra of each selected peak of chromatogram by targeted mass.

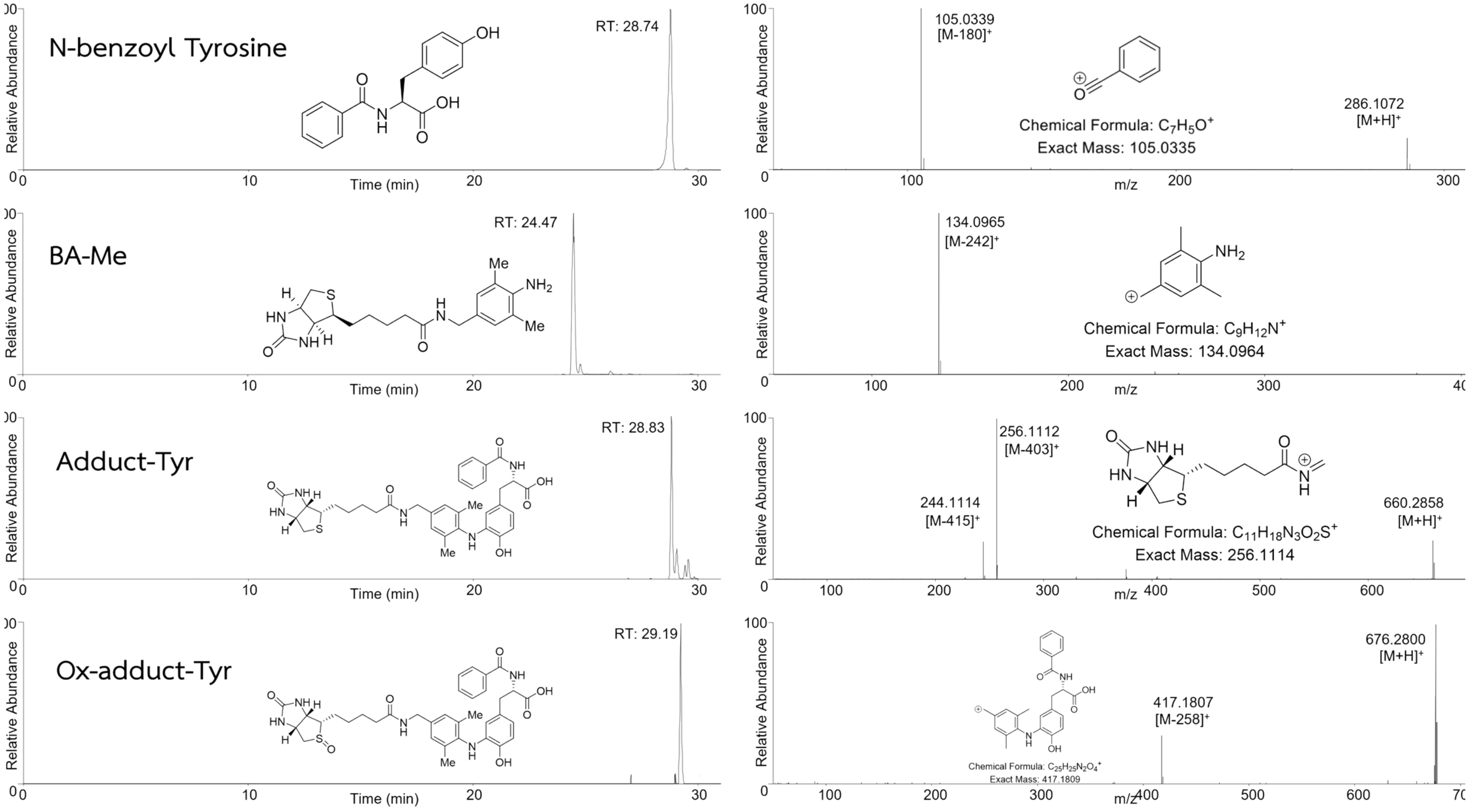

Figure S3. LC-MS/MS results of one-by-one adduct identification from horseradish peroxidase-mediated labeling reaction between **BA-Me** and *N*-benzoyl tyrosine. For each row of adduct, (Left) Chromatogram, (Right) Mass spectra of each selected peak of chromatogram by targeted mass.

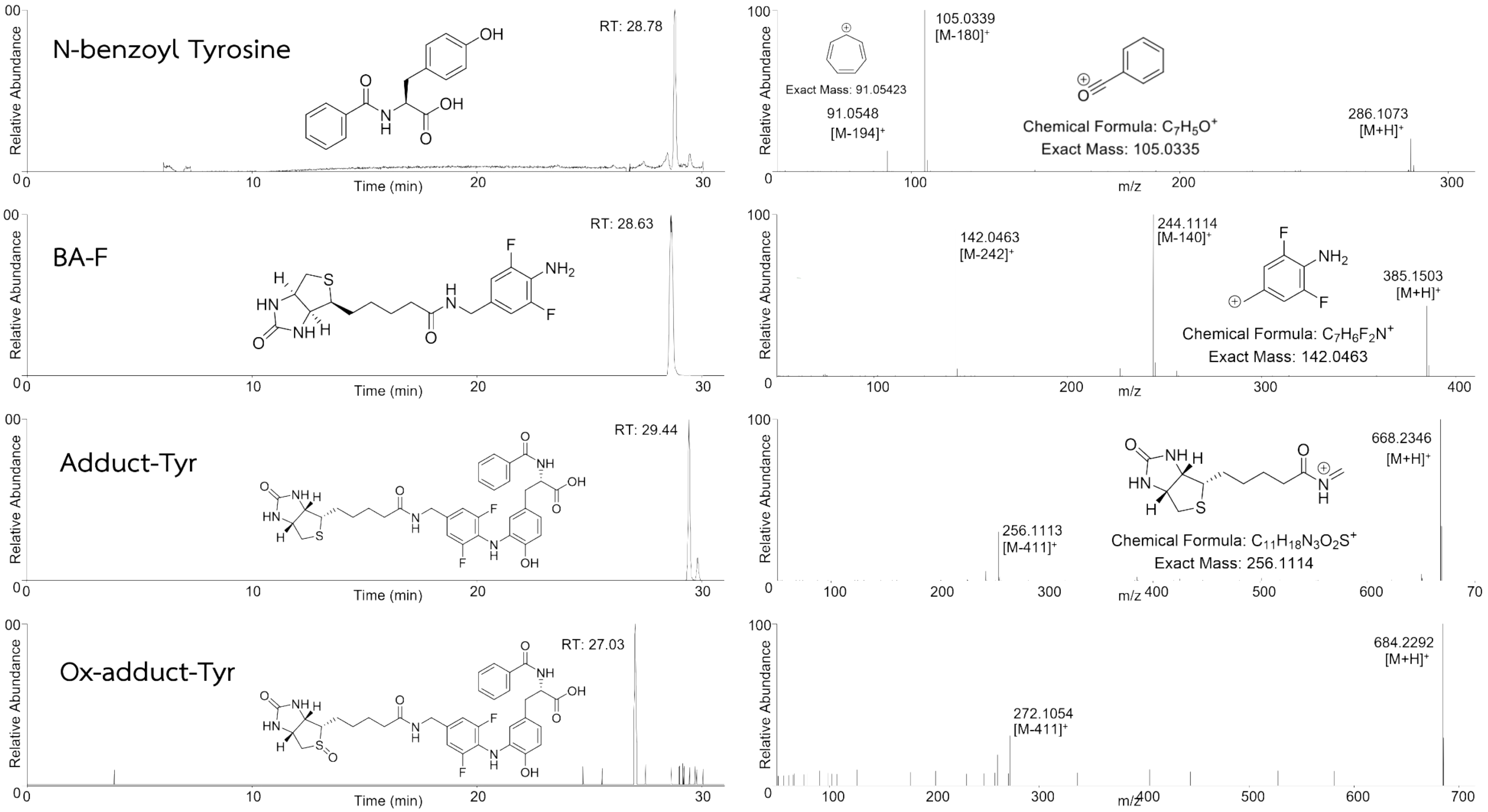

Figure S4. LC-MS/MS results of one-by-one adduct identification from horseradish peroxidase-mediated labeling reaction between **BA-F** and *N*-benzoyl tyrosine. For each row of adduct, (Left) Chromatogram, (Right) Mass spectra of each selected peak of chromatogram by targeted mass.

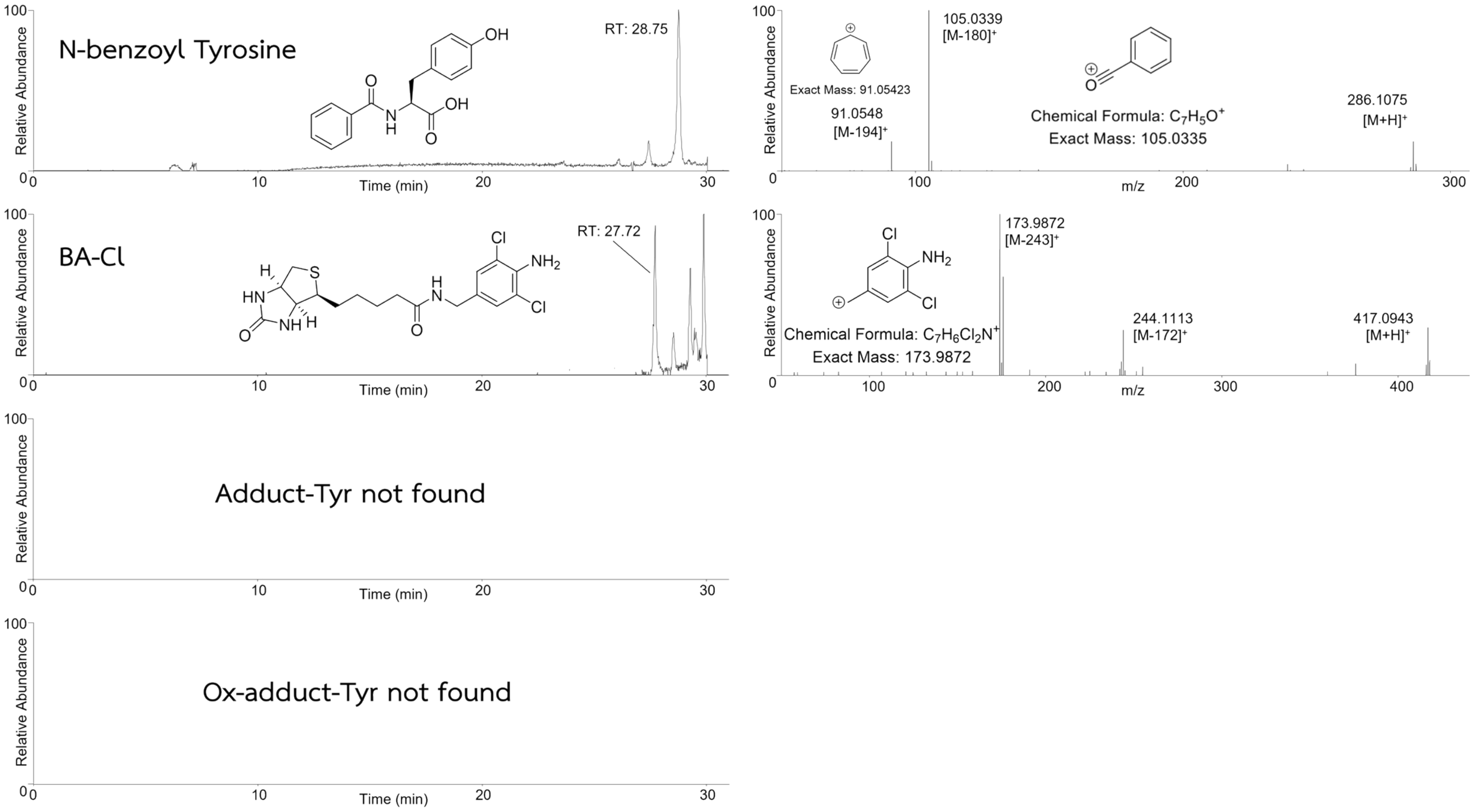

Figure S5. LC-MS/MS results of one-by-one adduct identification from horseradish peroxidase-mediated labeling reaction between **BA-Cl** and *N*-benzoyl tyrosine. For each row of adduct, (Left) Chromatogram, (Right) Mass spectra of each selected peak of chromatogram by targeted mass.

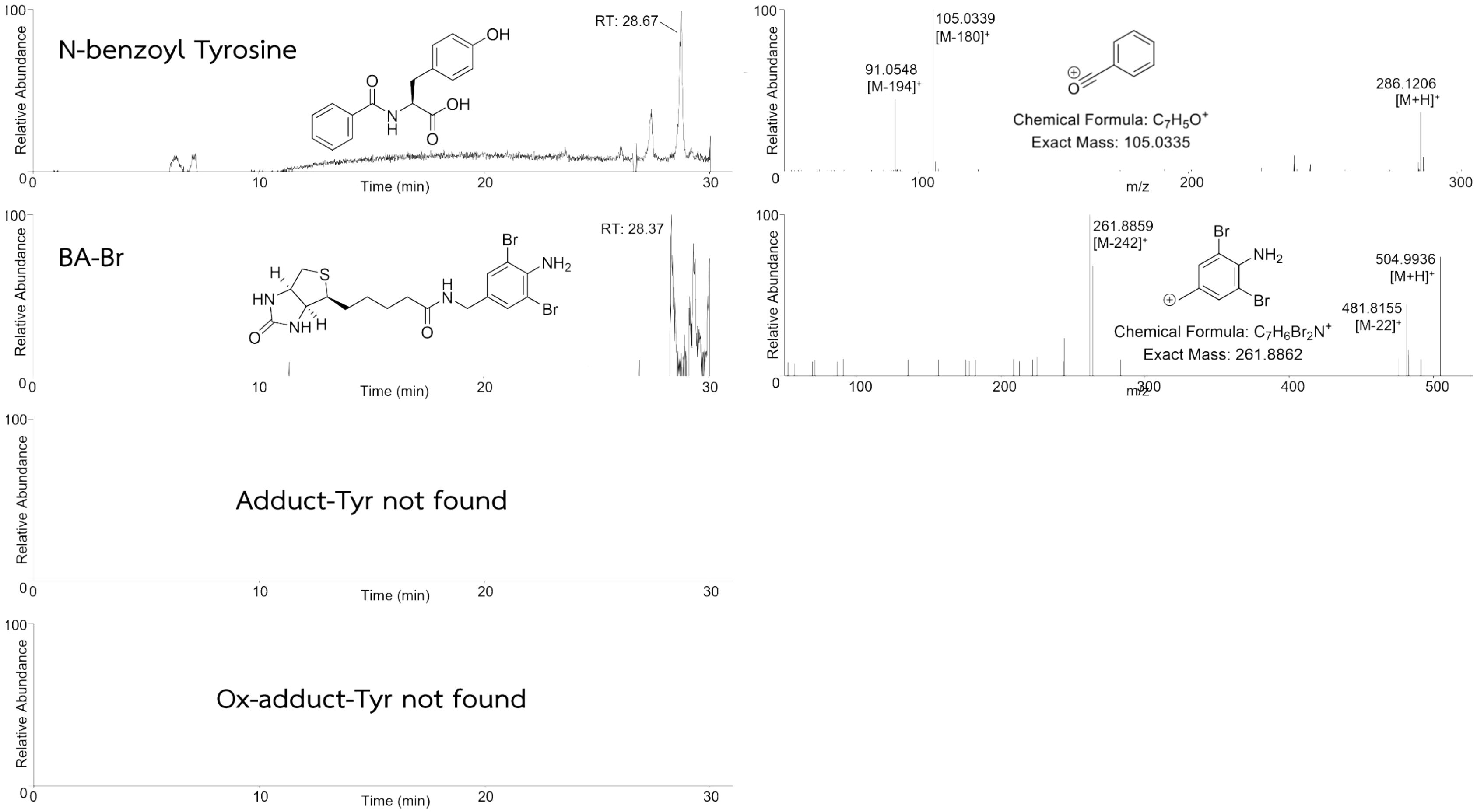

Figure S6. LC-MS/MS results of one-by-one adduct identification from horseradish peroxidase-mediated labeling reaction between **BA-Br** and *N*-benzoyl tyrosine. For each row of adduct, (Left) Chromatogram, (Right) Mass spectra of each selected peak of chromatogram by targeted mass.

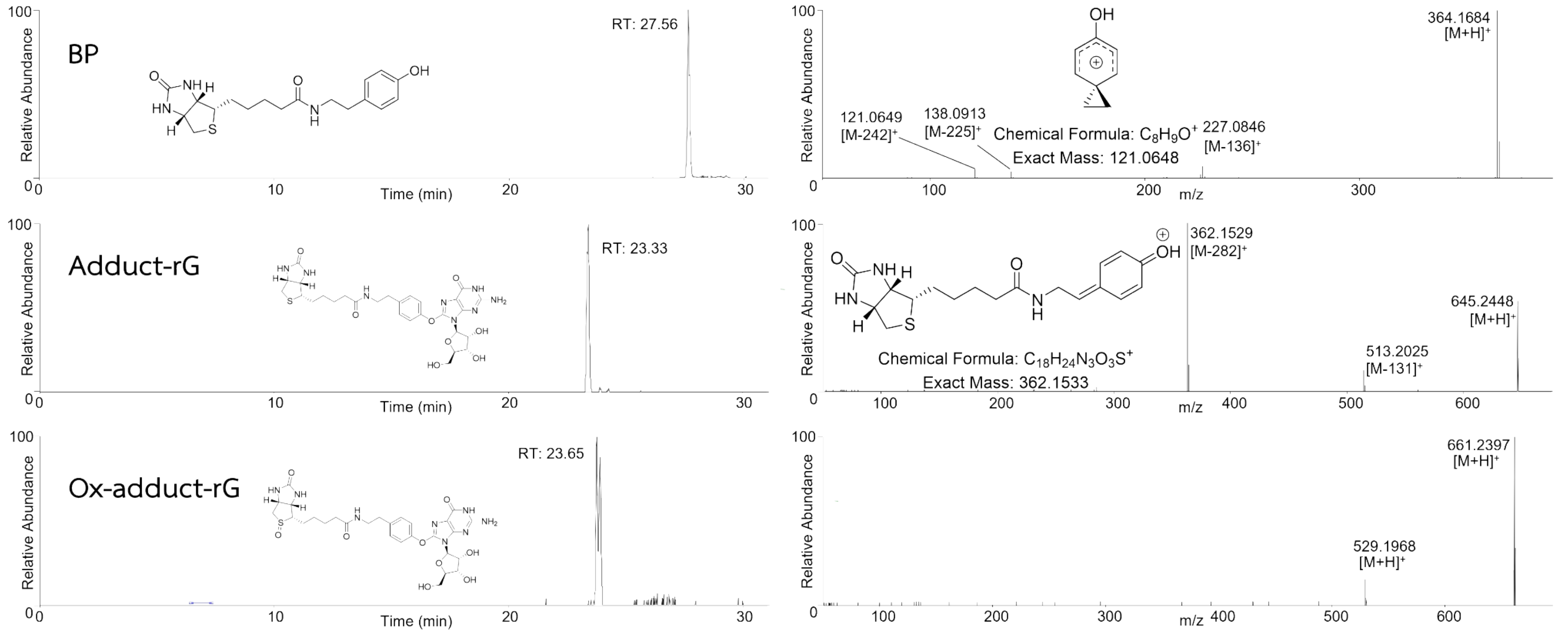

Figure S7. LC-MS/MS results of one-by-one adduct identification from horseradish peroxidase-mediated labeling reaction between **BP** and guanosine. For each row of adduct, (Left) Chromatogram, (Right) Mass spectra of each selected peak of chromatogram by targeted mass.

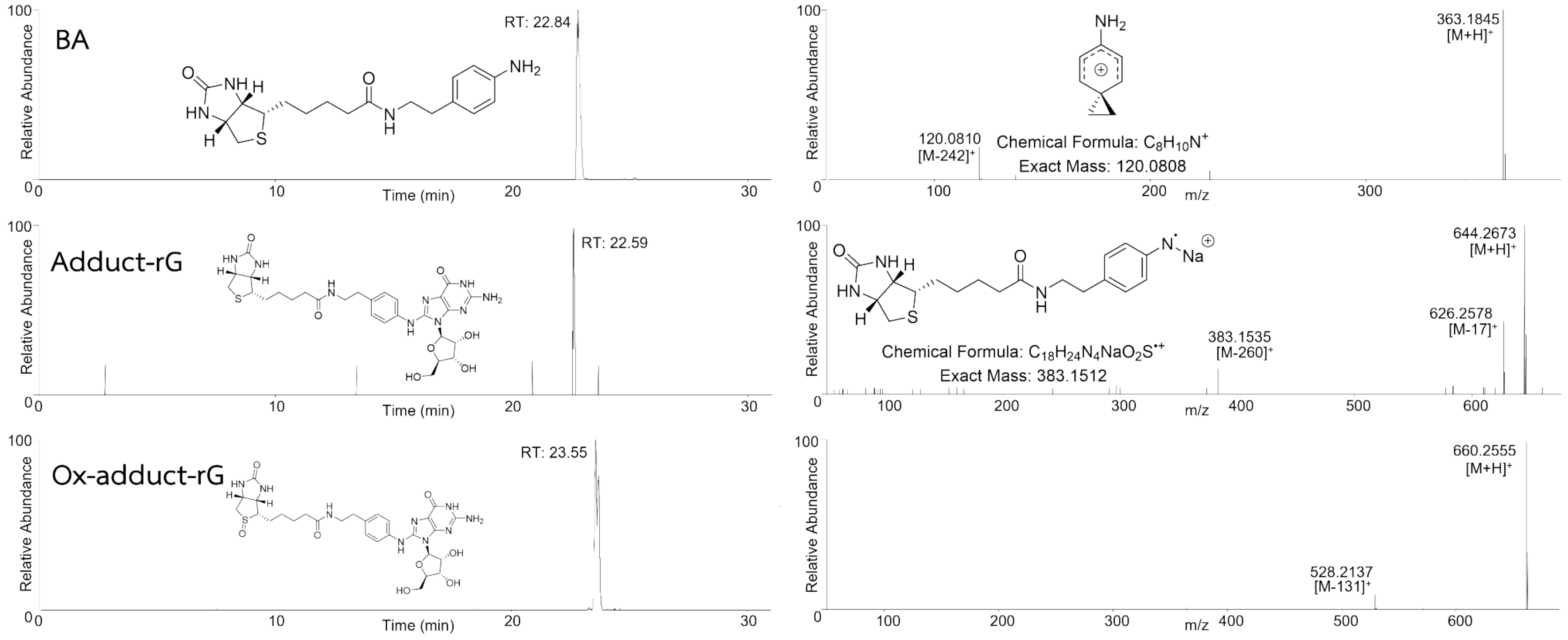

Figure S8. LC-MS/MS results of one-by-one adduct identification from horseradish peroxidase-mediated labeling reaction between **BA** and guanosine. For each row of adduct, (Left) Chromatogram, (Right) Mass spectra of each selected peak of chromatogram by targeted mass.

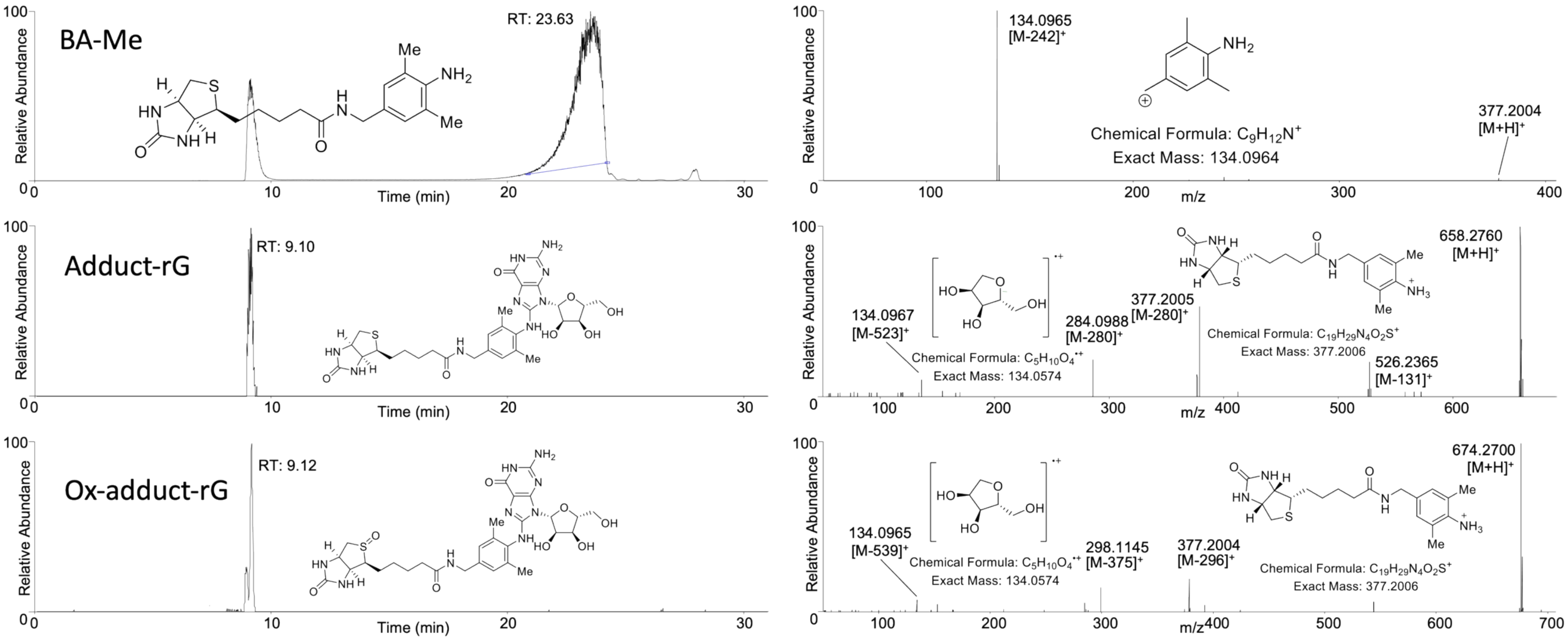

Figure S9. LC-MS/MS results of one-by-one adduct identification from horseradish peroxidase-mediated labeling reaction between **BA-Me** and guanosine. For each row of adduct, (Left) Chromatogram, (Right) Mass spectra of each selected peak of chromatogram by targeted mass.

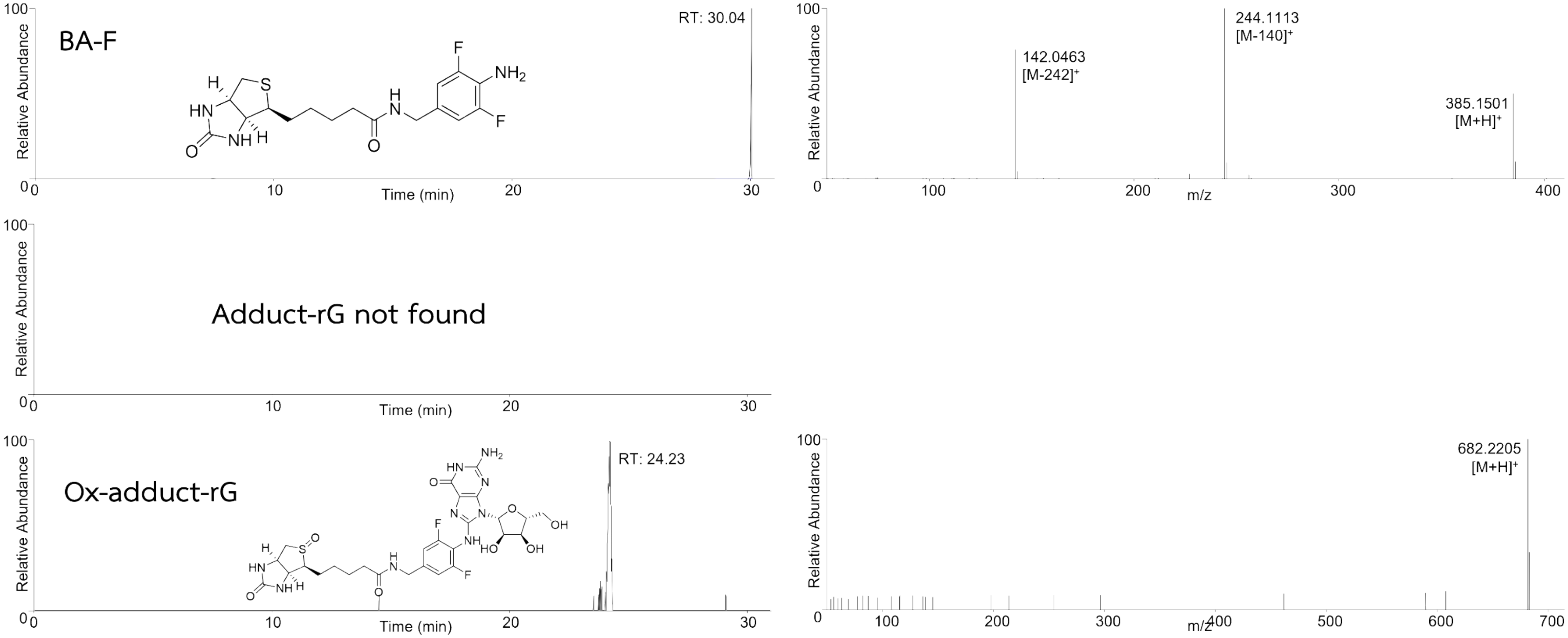

Figure S10. LC-MS/MS results of one-by-one adduct identification from horseradish peroxidase-mediated labeling reaction between **BA-F** and guanosine. For each row of adduct, (Left) Chromatogram, (Right) Mass spectra of each selected peak of chromatogram by targeted mass.

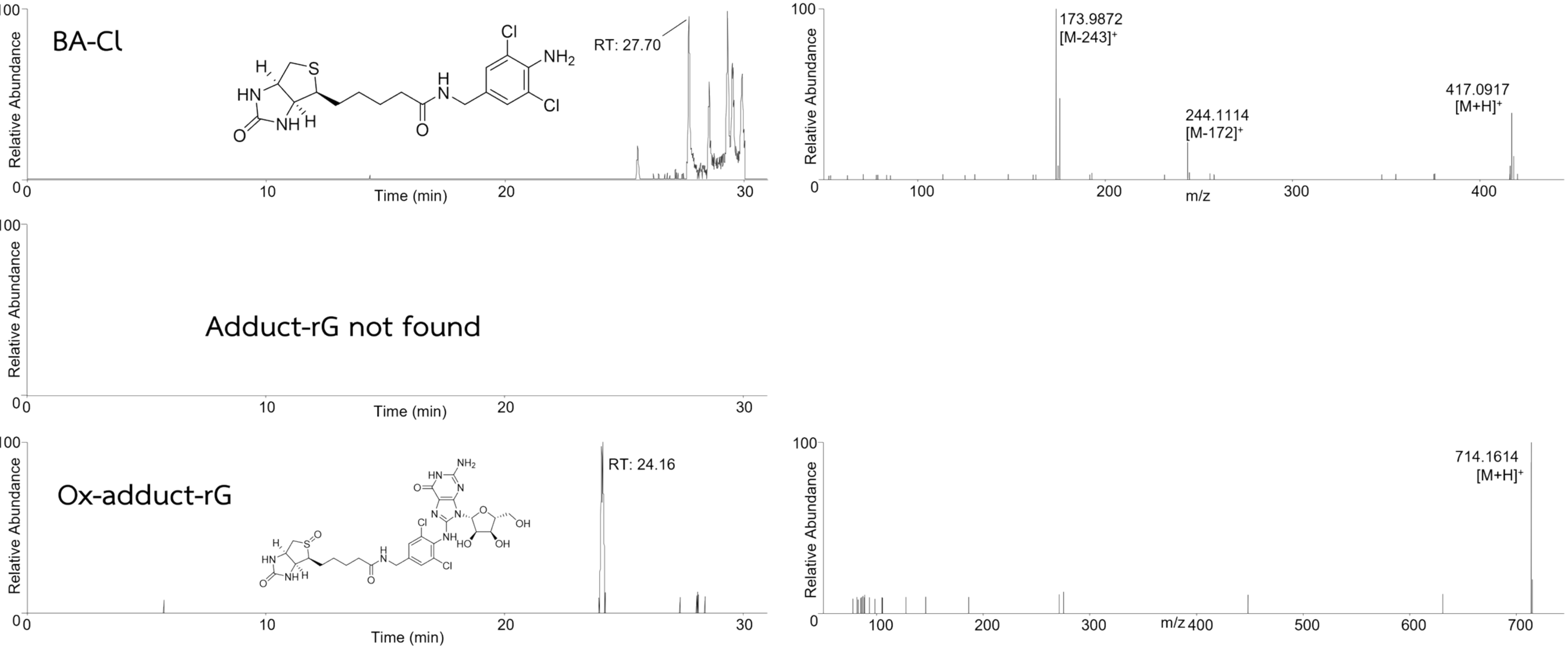

Figure S11. LC-MS/MS results of one-by-one adduct identification from horseradish peroxidase-mediated labeling reaction between **BA-Cl** and guanosine. For each row of adduct, (Left) Chromatogram, (Right) Mass spectra of each selected peak of chromatogram by targeted mass.

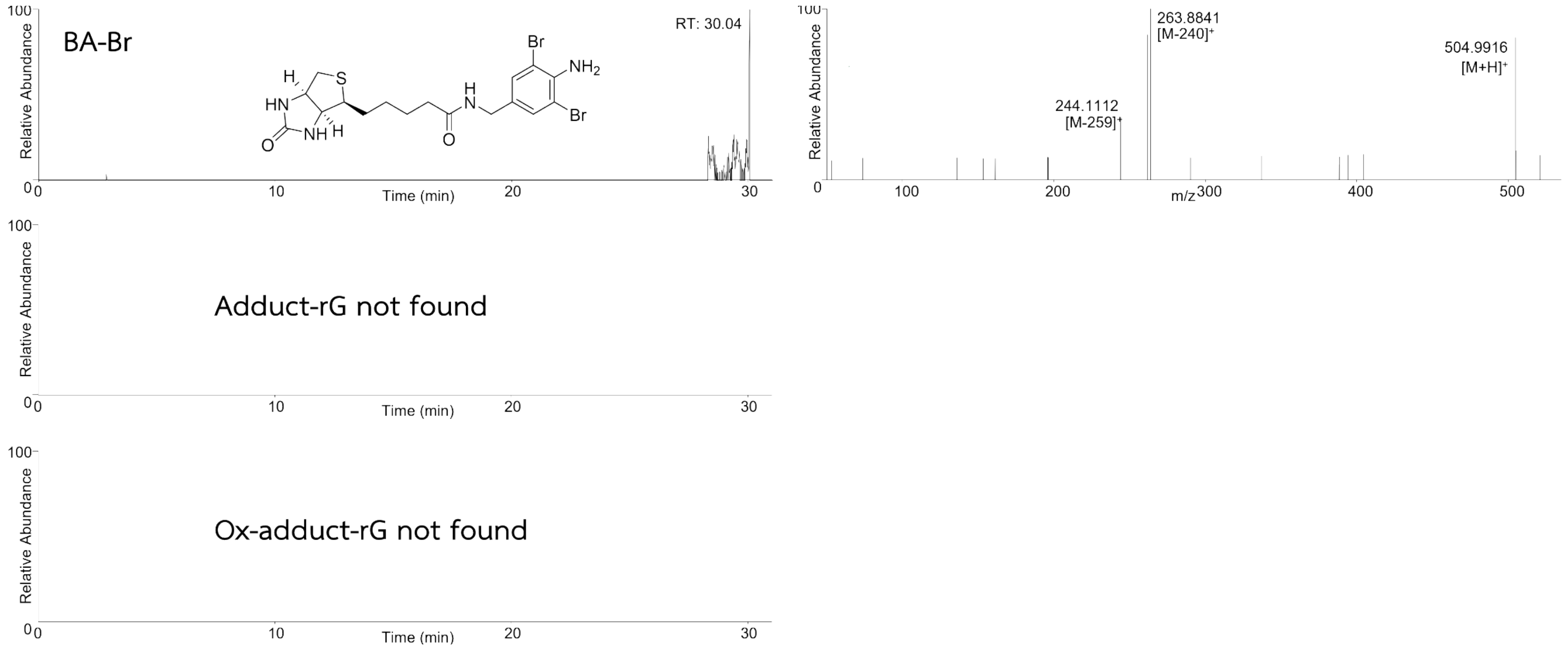

Figure S12. LC-MS/MS results of one-by-one adduct identification from horseradish peroxidase-mediated labeling reaction between **BA-Br** and guanosine. For each row of adduct, (Left) Chromatogram, (Right) Mass spectra of each selected peak of chromatogram by targeted mass.

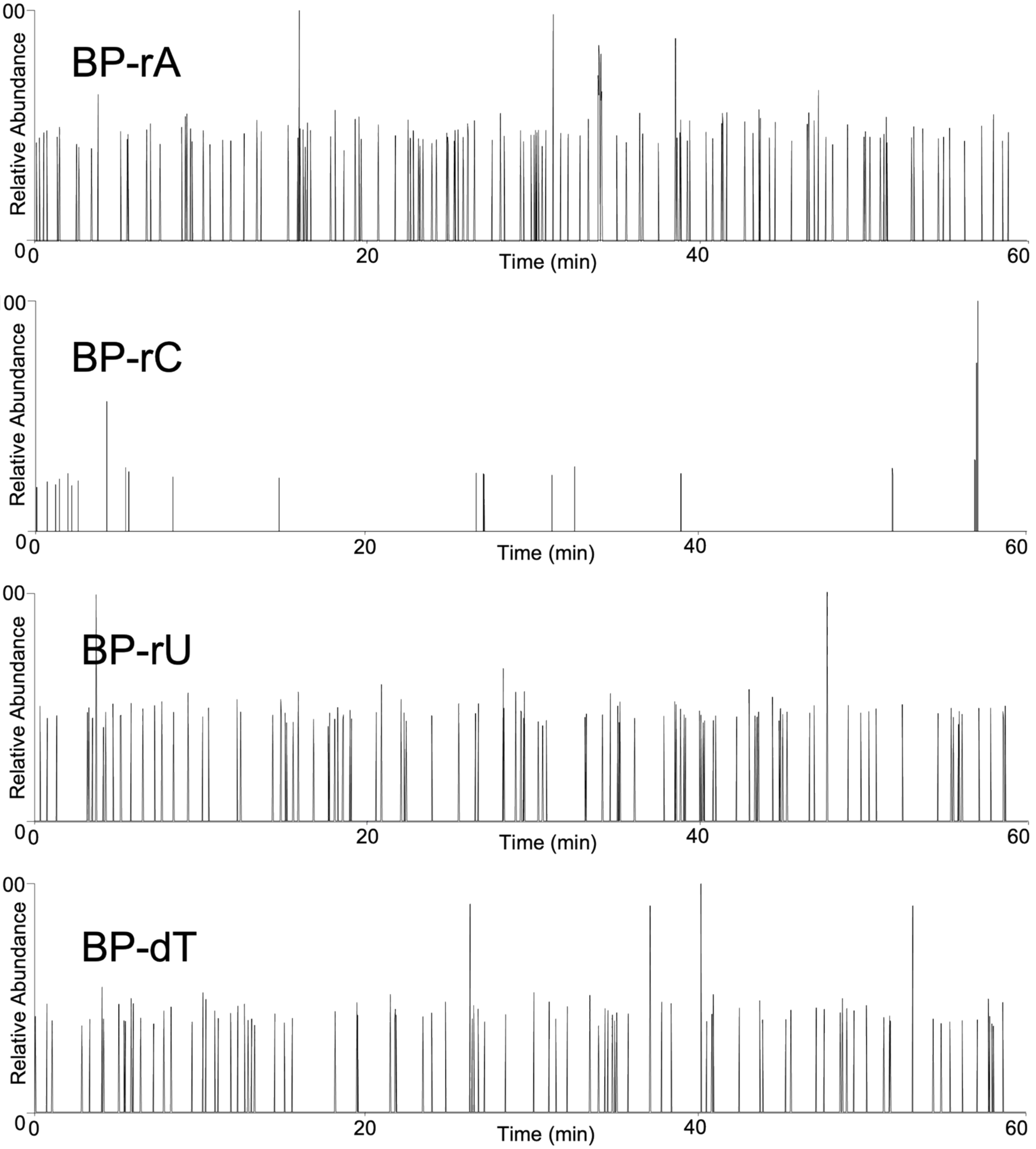

Figure S13. LC-MS/MS results of one-by-one adduct identification from horseradish peroxidase-mediated labeling reaction between **BP** and adenosine (rA), cytosine (rC), uridine (rU), and thymidine (dT). No adduct was found.

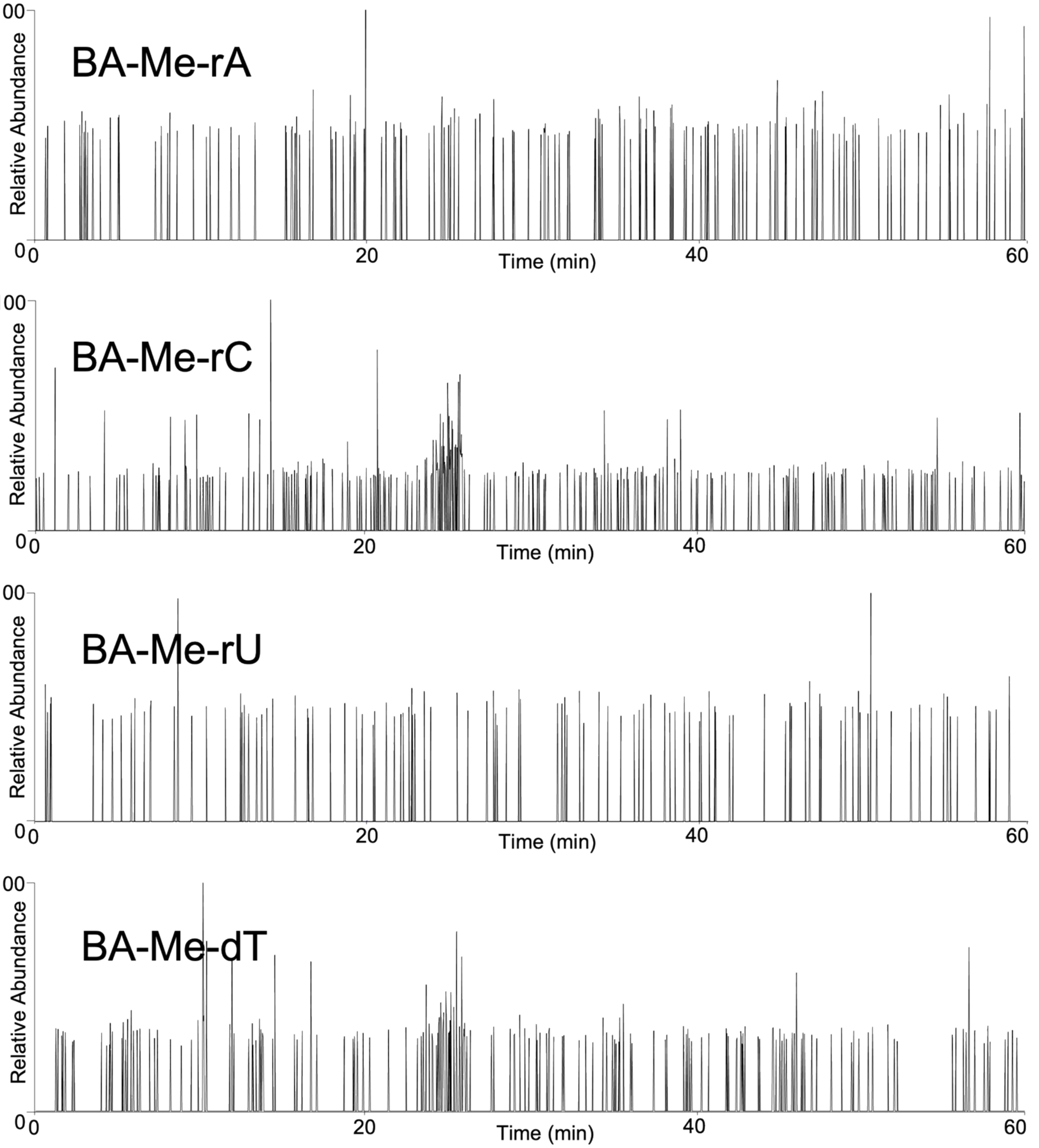

Figure S14. LC-MS/MS results of one-by-one adduct identification from horseradish peroxidase-mediated labeling reaction between **BA-Me** and adenosine (rA), cytosine (rC), uridine (rU), and thymidine (dT). No adduct was found.

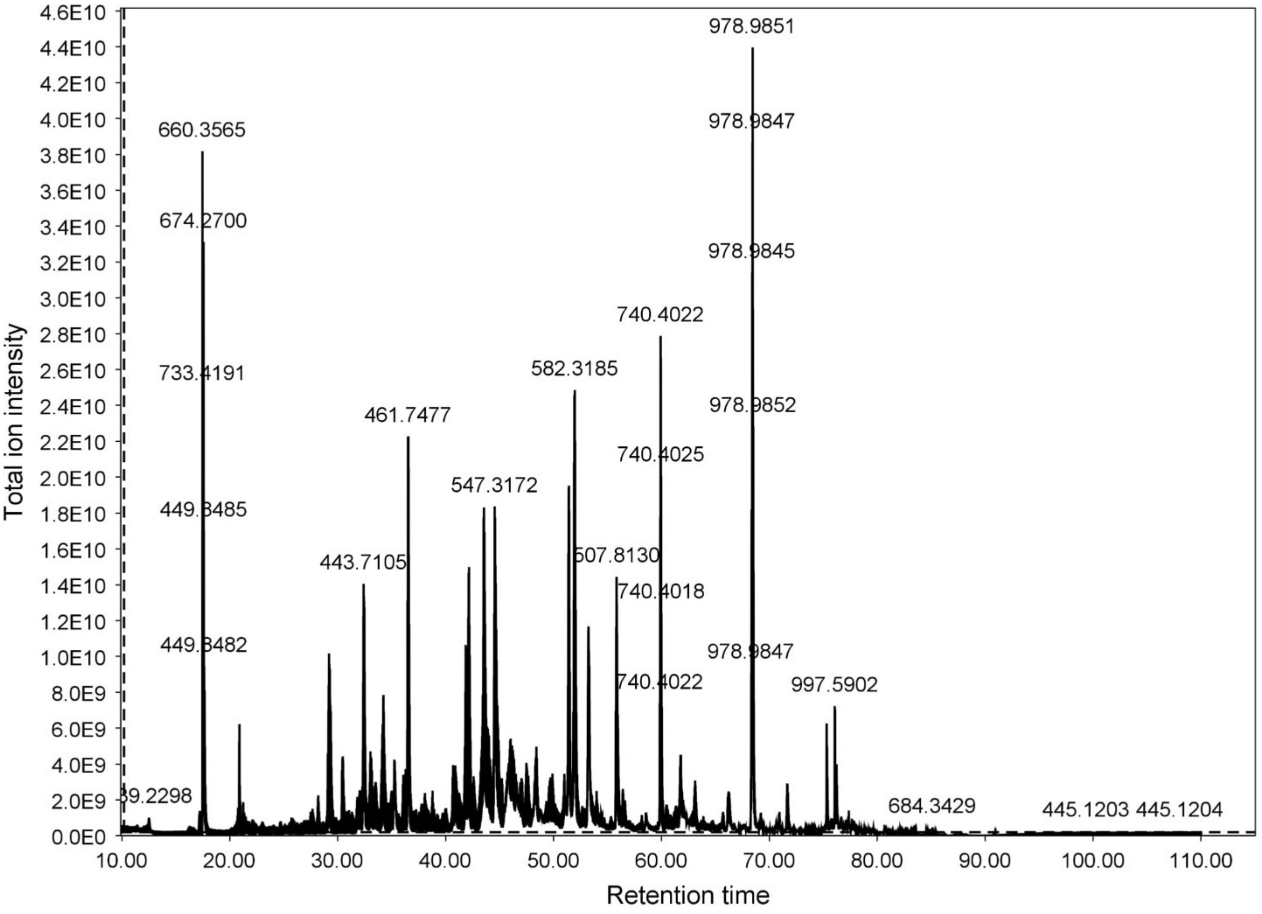

Figure S15. Chromatogram of lysed BSA labelled by **BP**

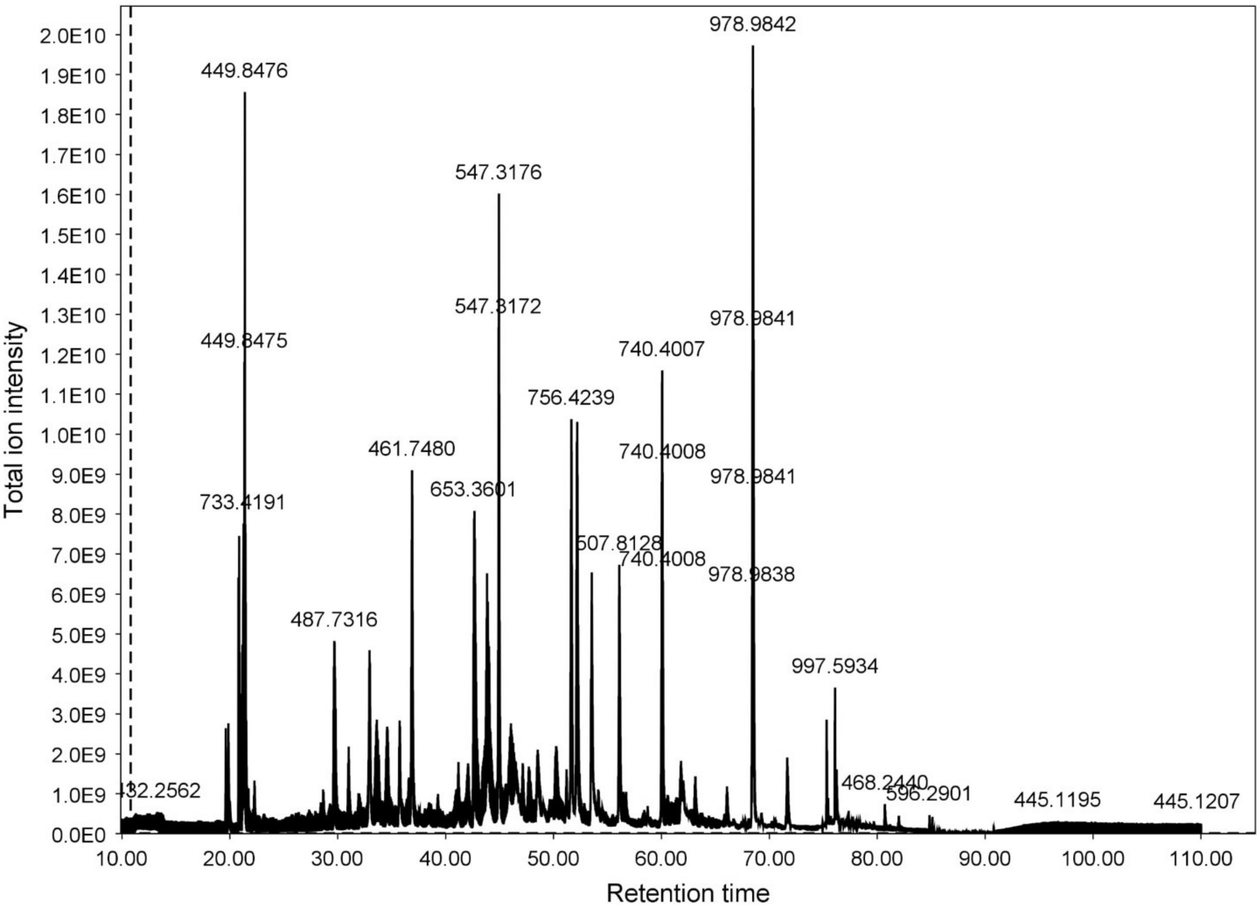

Figure S16. Chromatogram of lysed BSA labelled by **DBP**

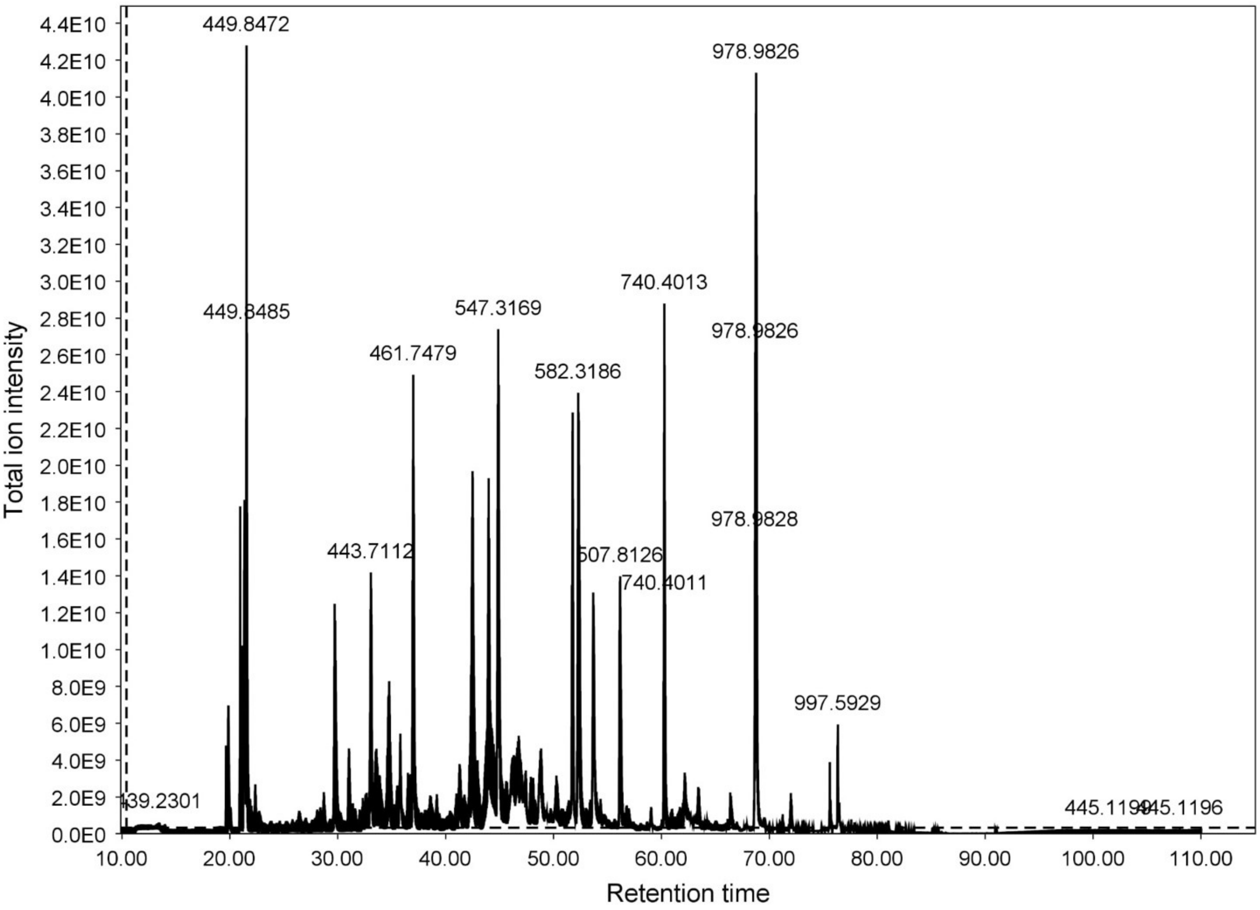

Figure S17. Chromatogram of lysed BSA labelled by **DBA-Me**

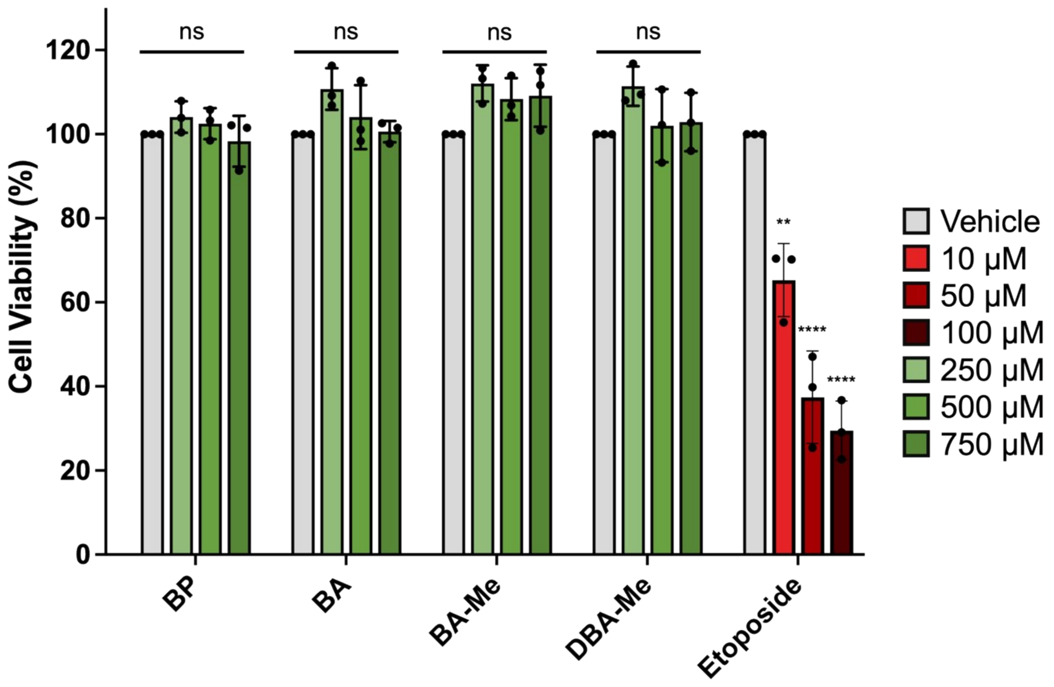

Figure S18. Cytotoxicity evaluation of desthiobiotin probes using MTT assay. HEK293FT cells were treated with different concentrations (250, 500, 750 μM) of each compound for 24 h. For control, the cells were treated with Etoposide, a chemotherapeutic drug. The results are shown as the mean ± standard deviation values of three independent experiments (n=3) evaluated by one-way ANOVA (a = 0.05): ns, not significant; **, $p < 0.005$; ****, $p < 0.0001$ vs vehicle (control).

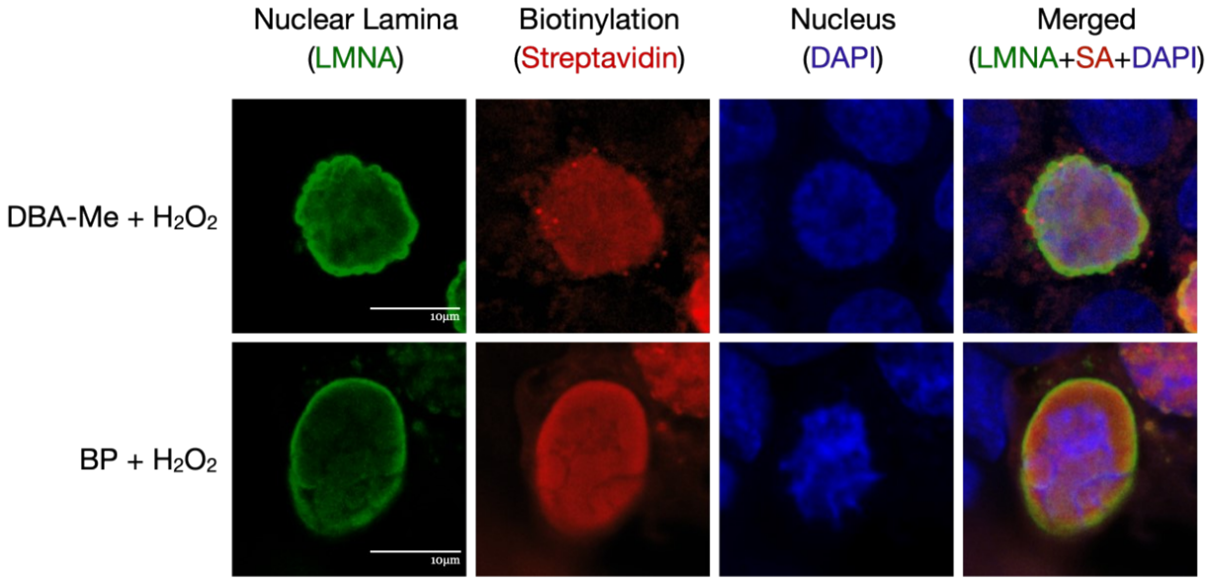

Figure S19. Immunofluorescence of APEX2-mediated biotinylation. HEK293FT cells expressing LMNA-V5-APEX2 were biotinylated, fixed, and stained as described in experimental section. Row 1 and 2 show the cells treated with **DBA-Me** and **BP** in the presence of H_2O_2, respectively. Scale bars, 10 μm. SA = Streptavidin.

NMR Spectra

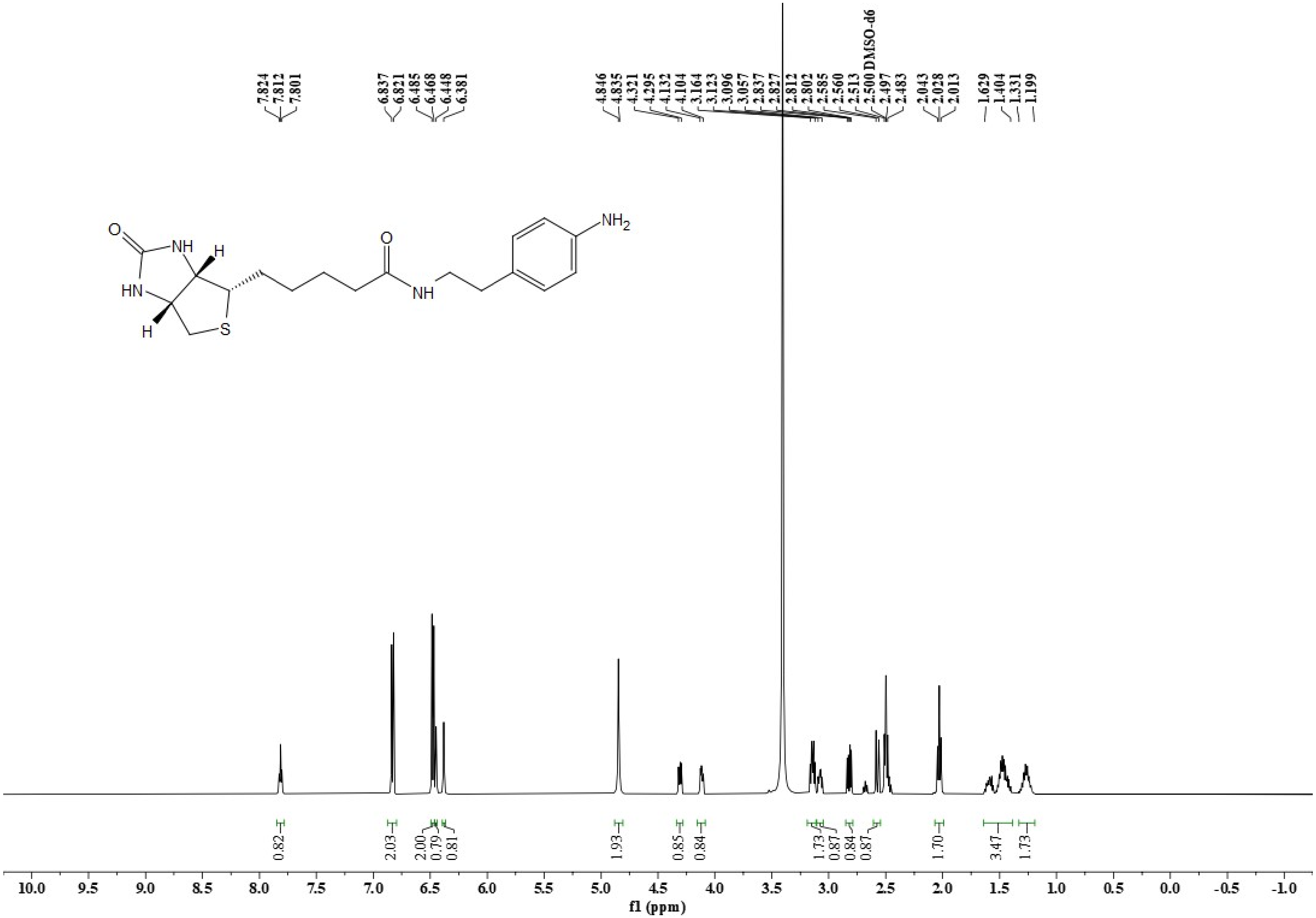

Figure S20. ^{1}H NMR spectrum of **BA** in DMSO-d_6 (500 MHz).

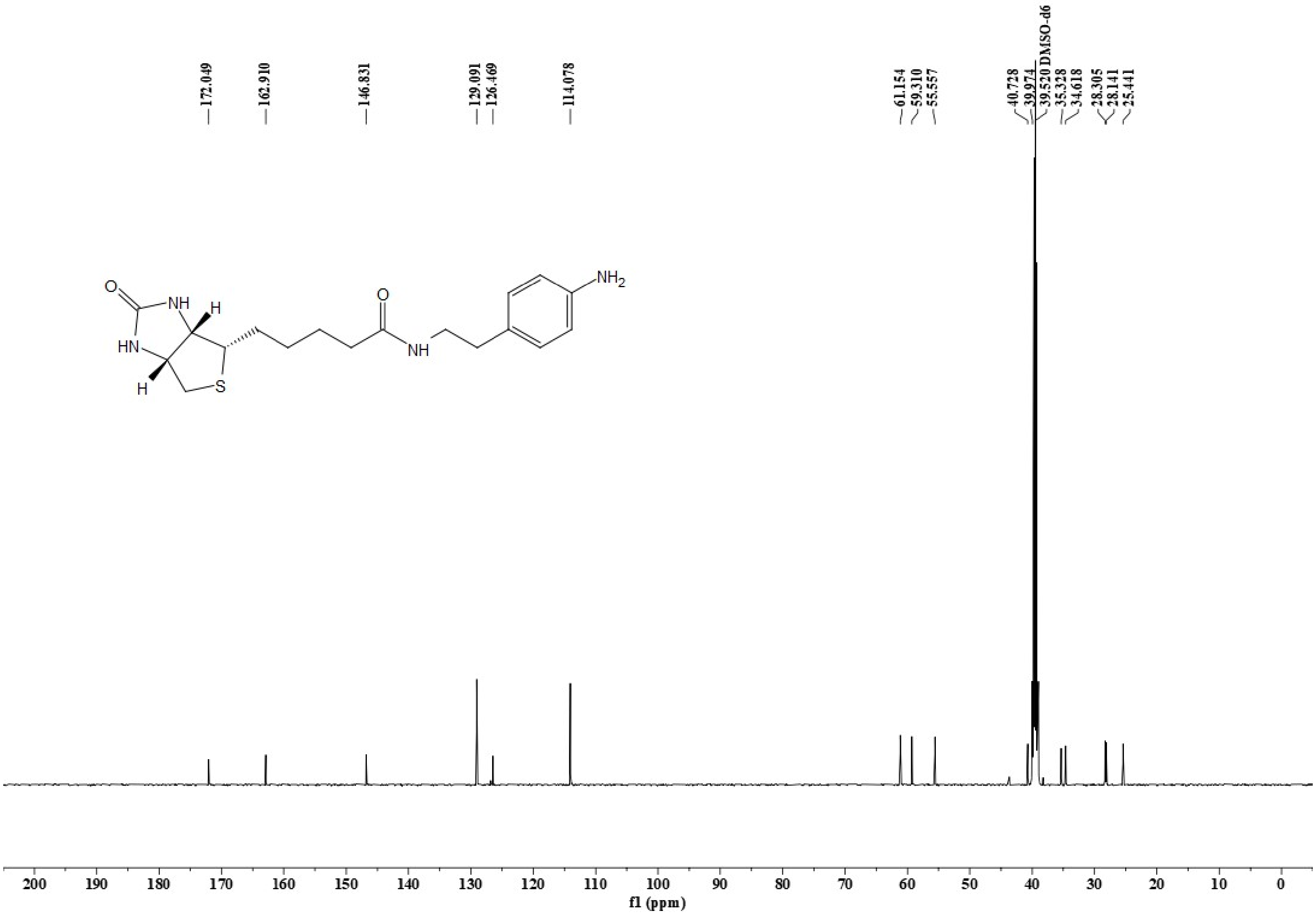

Figure S21. ^{13}C NMR spectrum of **BA** in DMSO-d_6 (126 MHz).

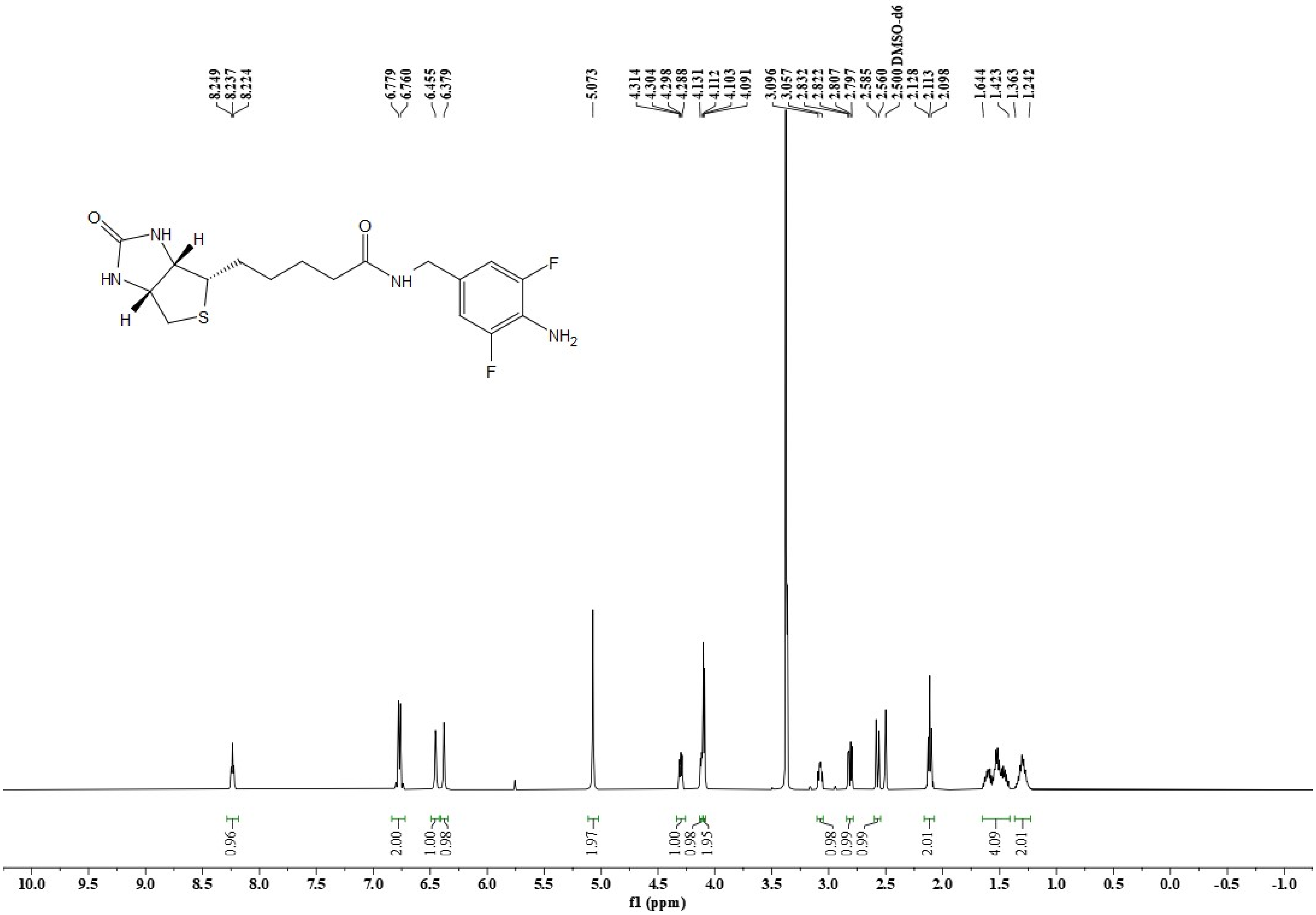

Figure S22. ^{1}H NMR spectrum of **BA-F** in DMSO-d_6 (500 MHz).

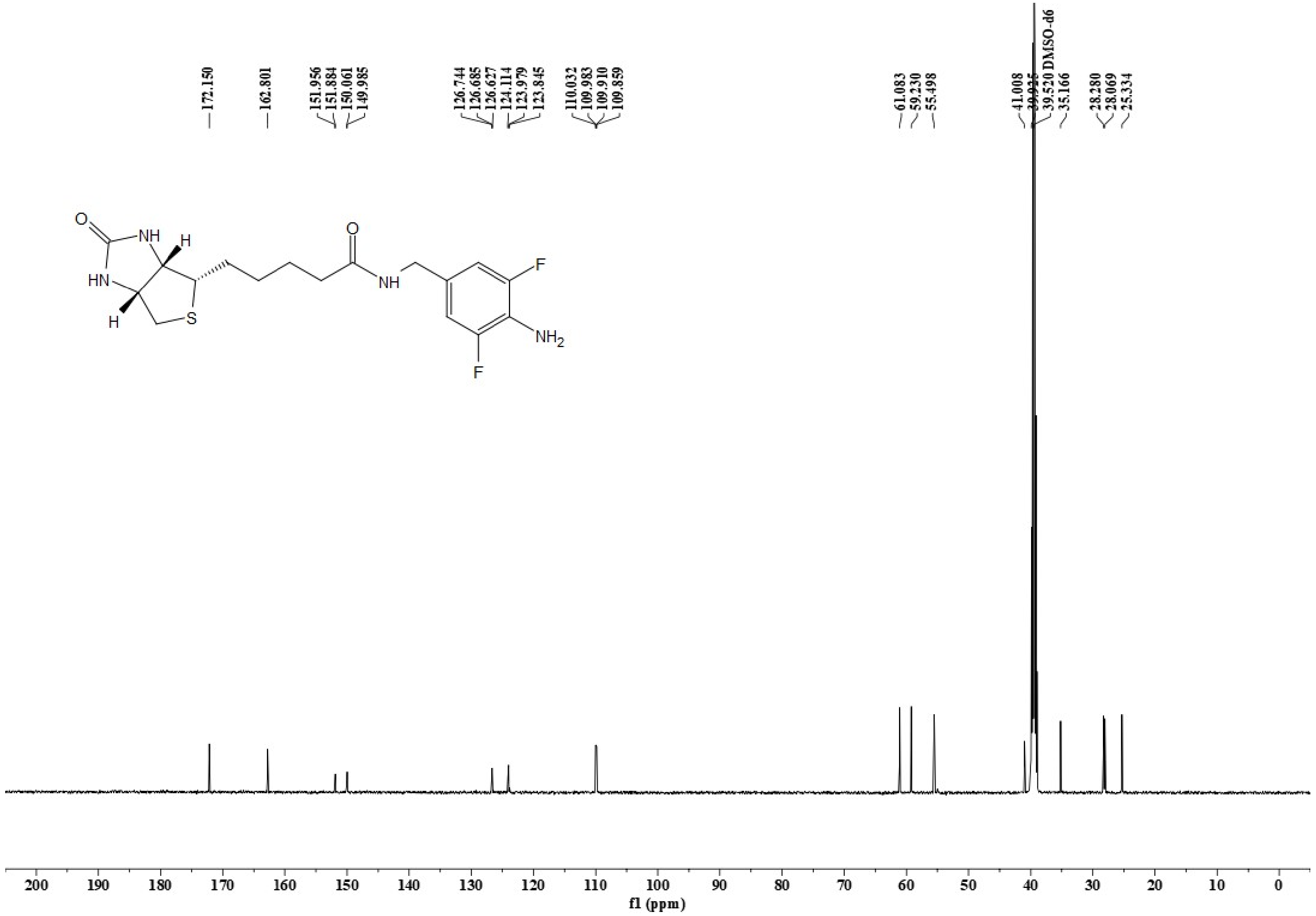

Figure S23. ^{13}C NMR spectrum of **BA-F** in DMSO-d_6 (126 MHz).

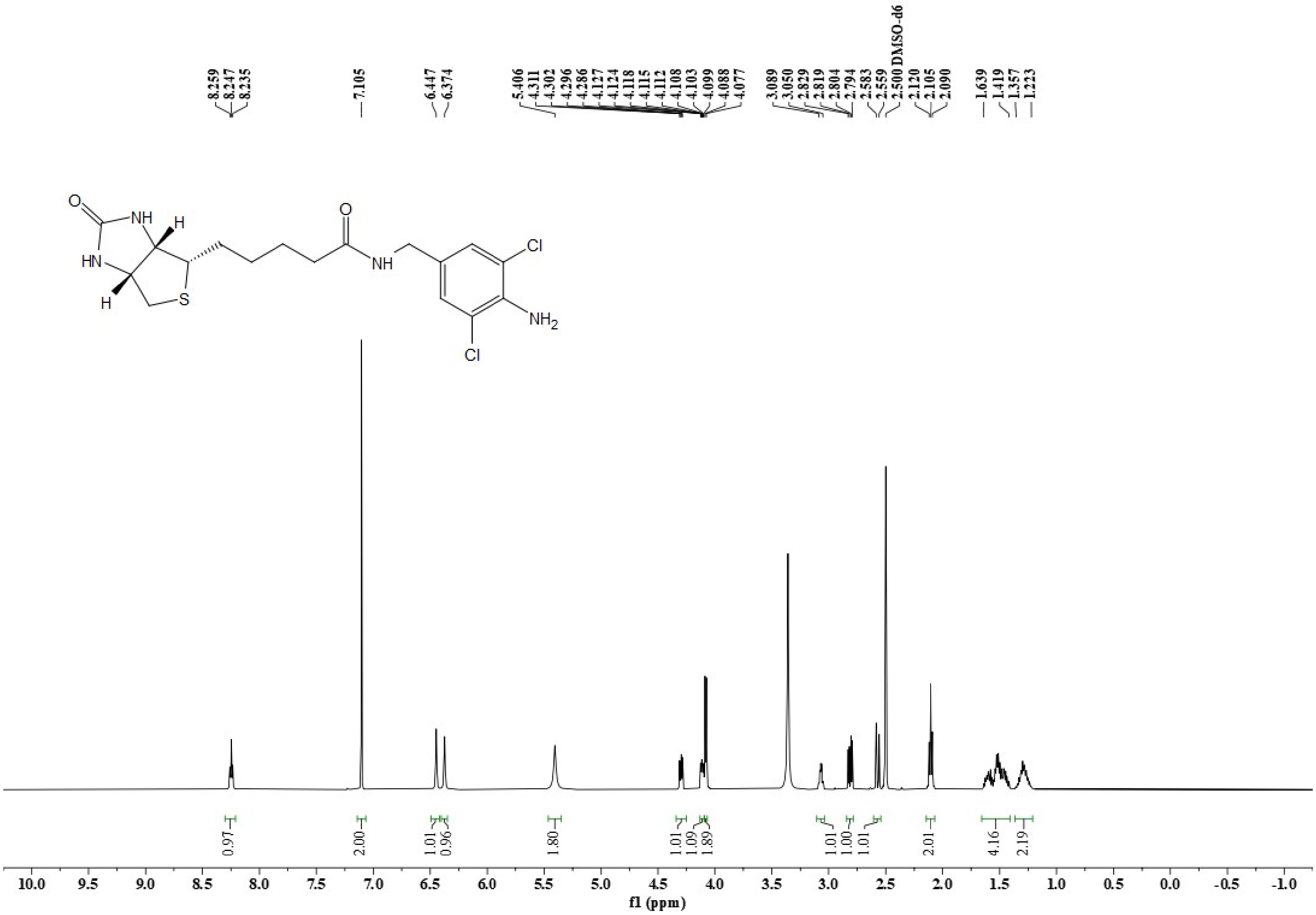

Figure S24. ^{1}H NMR spectrum of **BA-Cl** in DMSO-d_6 (500 MHz).

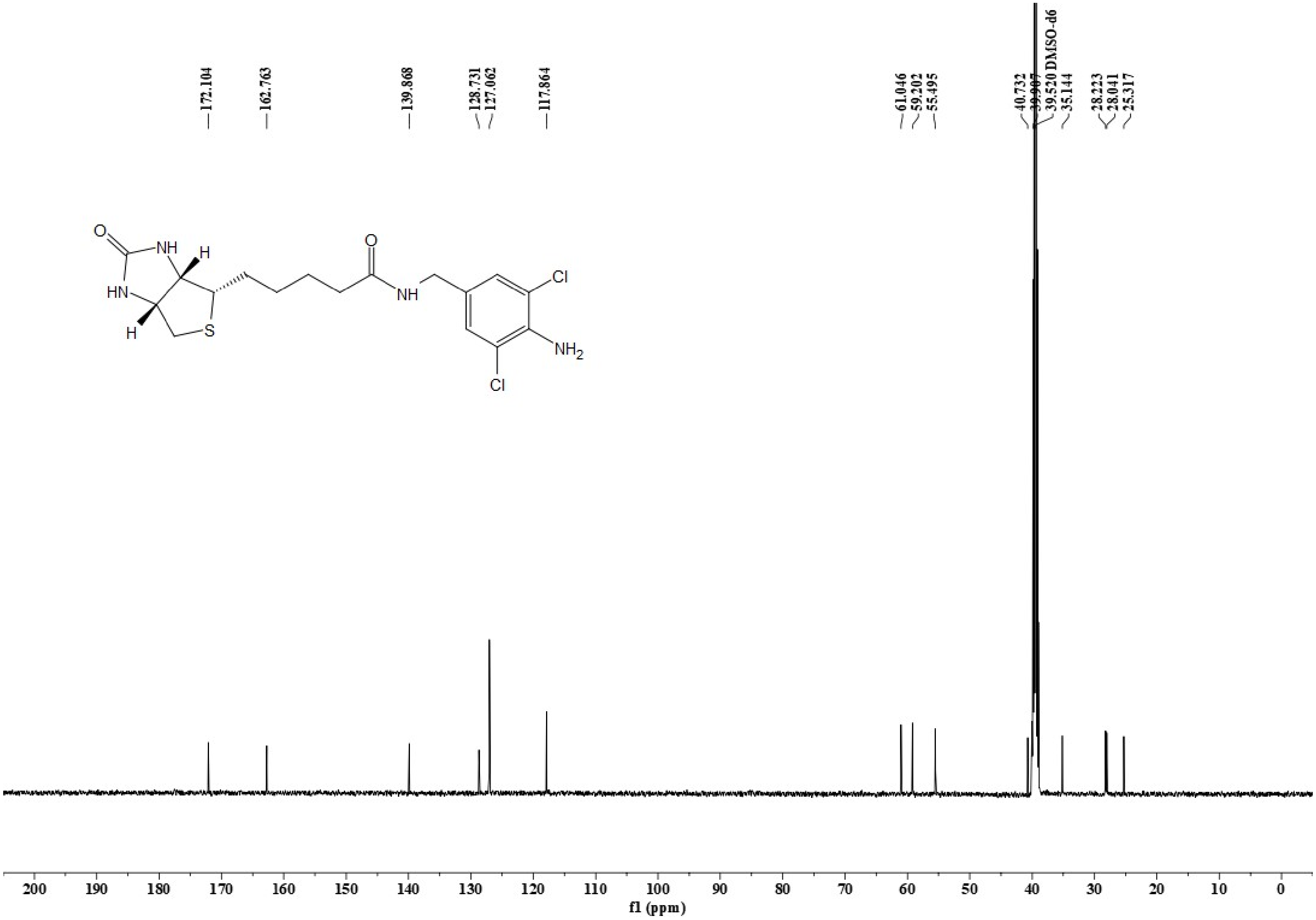

Figure S25. ^{13}C NMR spectrum of **BA-Cl** in DMSO-d_6 (126 MHz).

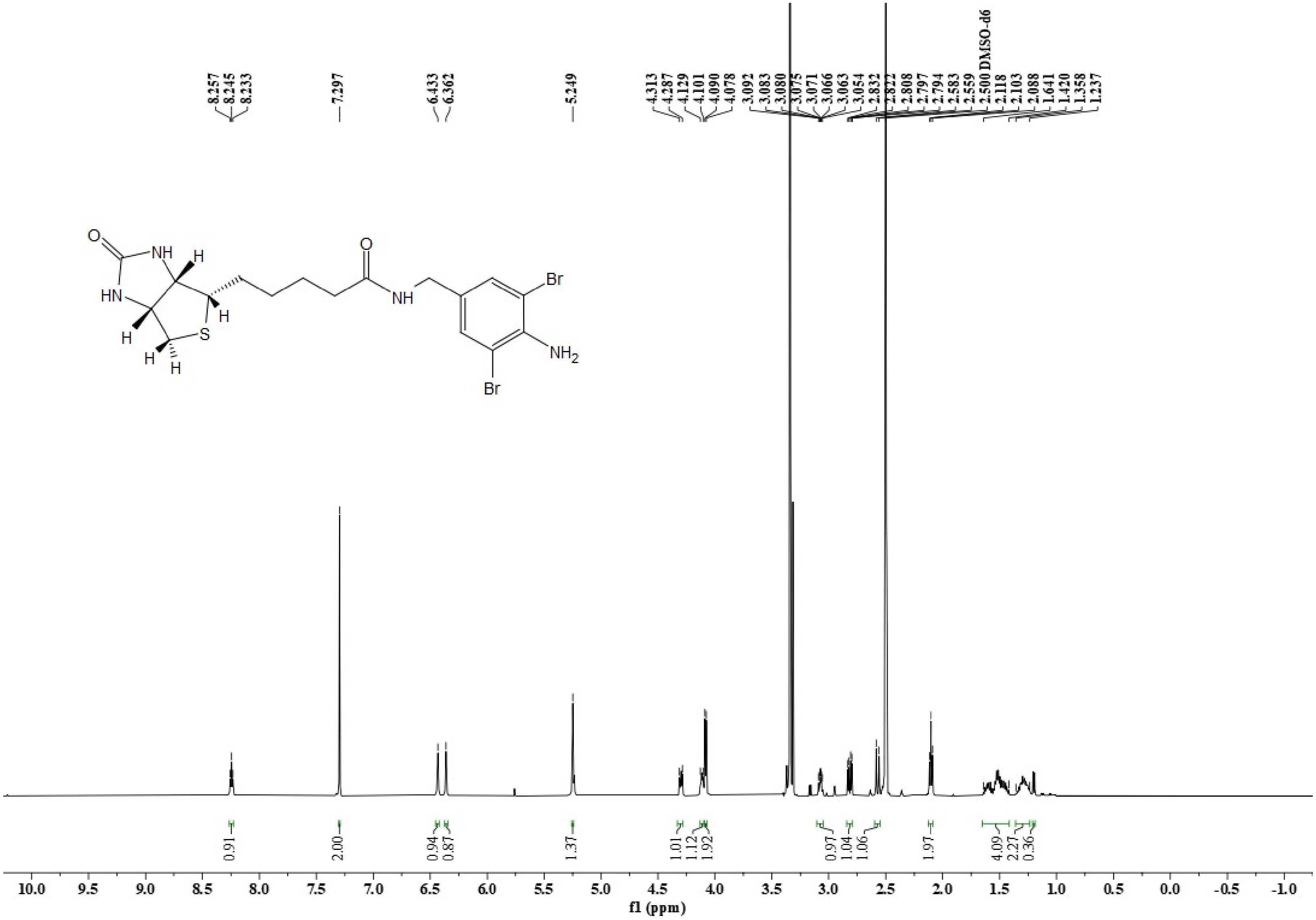

Figure S26. ^1H NMR spectrum of **BA-Br** in DMSO-d_6 (500 MHz).

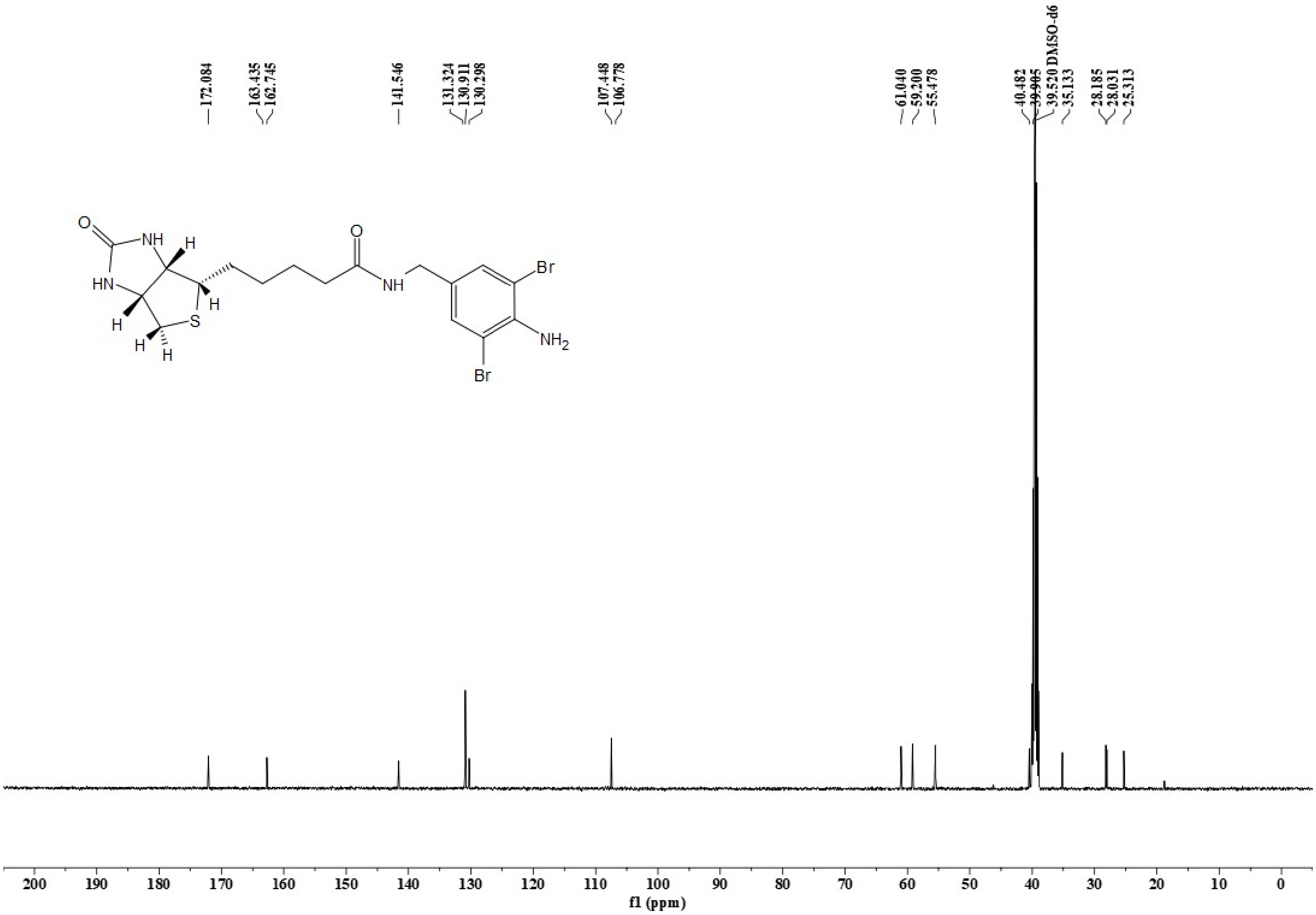

Figure S27. ^{13}C NMR spectrum of **BA-Br** in DMSO-d_6 (126 MHz).

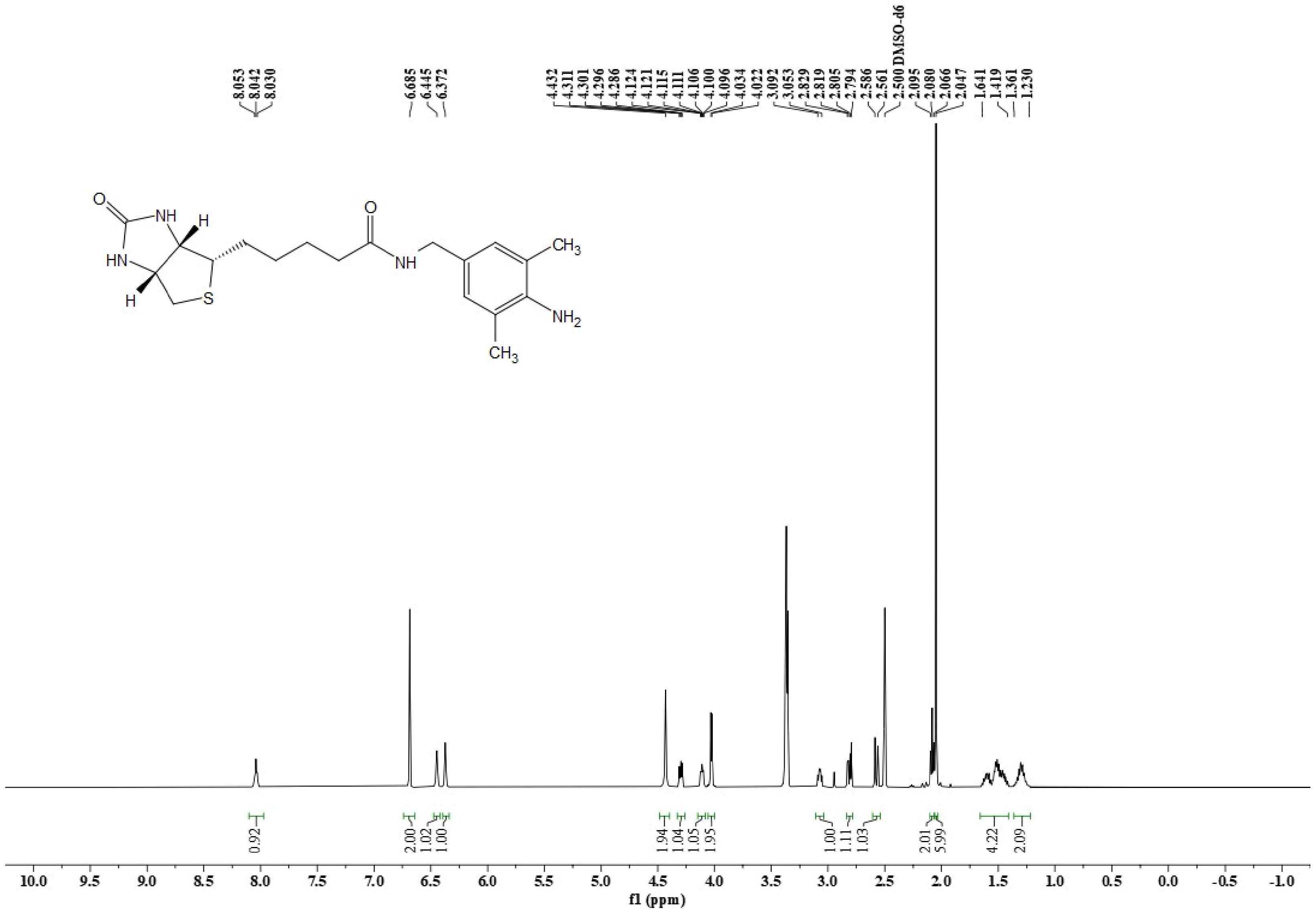

Figure S28. ^{1}H NMR spectrum of **BA-Me** in DMSO-d_6 (500 MHz).

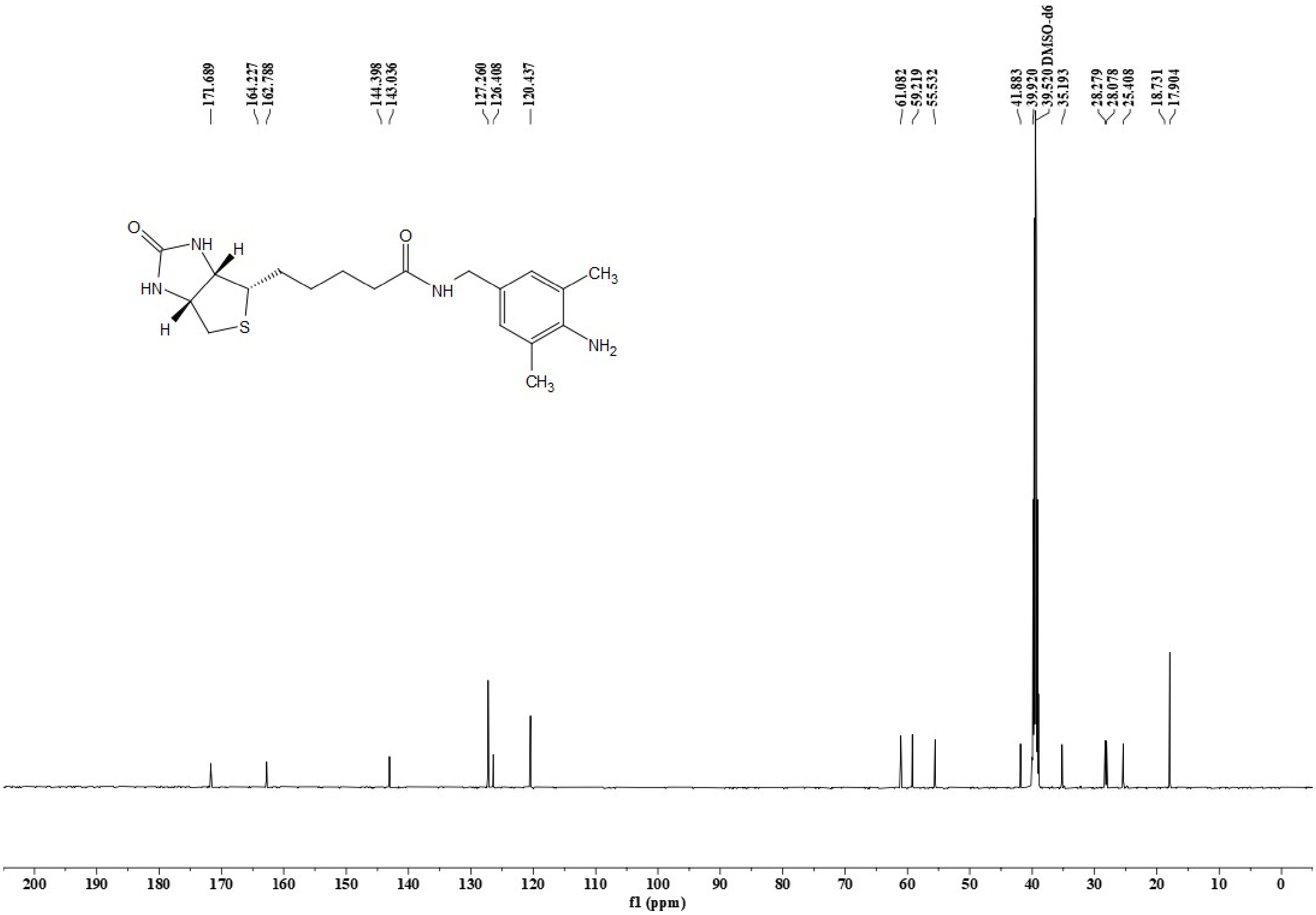

Figure S29. ^{13}C NMR spectrum of **BA-Me** in DMSO-d_6 (126 MHz).

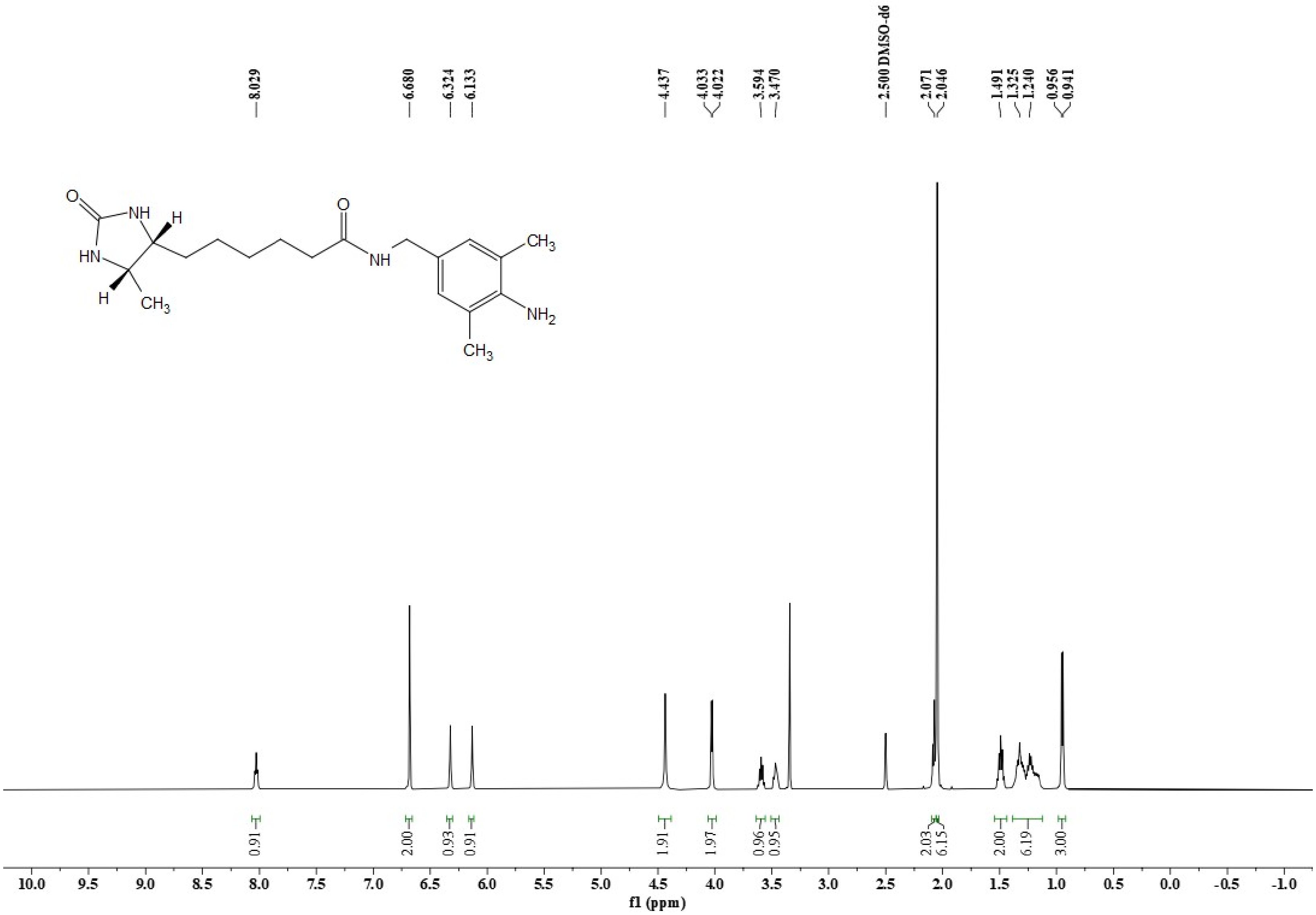

Figure S30. ^{1}H NMR spectrum of **DBA-Me** in DMSO-d_6 (500 MHz).

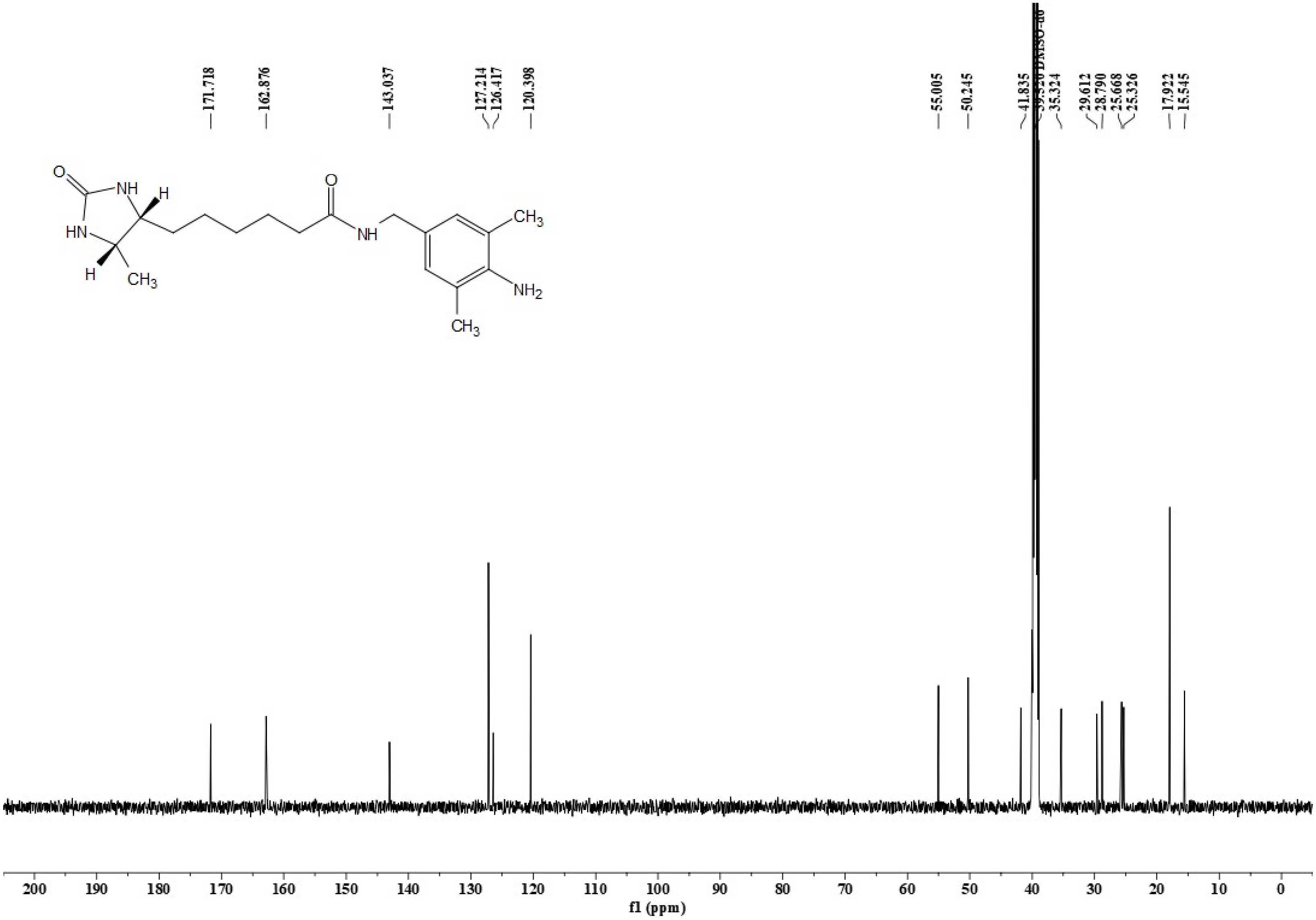

Figure S31. ^{13}C NMR spectrum of **DBA-Me** in DMSO-d_6 (126 MHz).

Raw Image Data

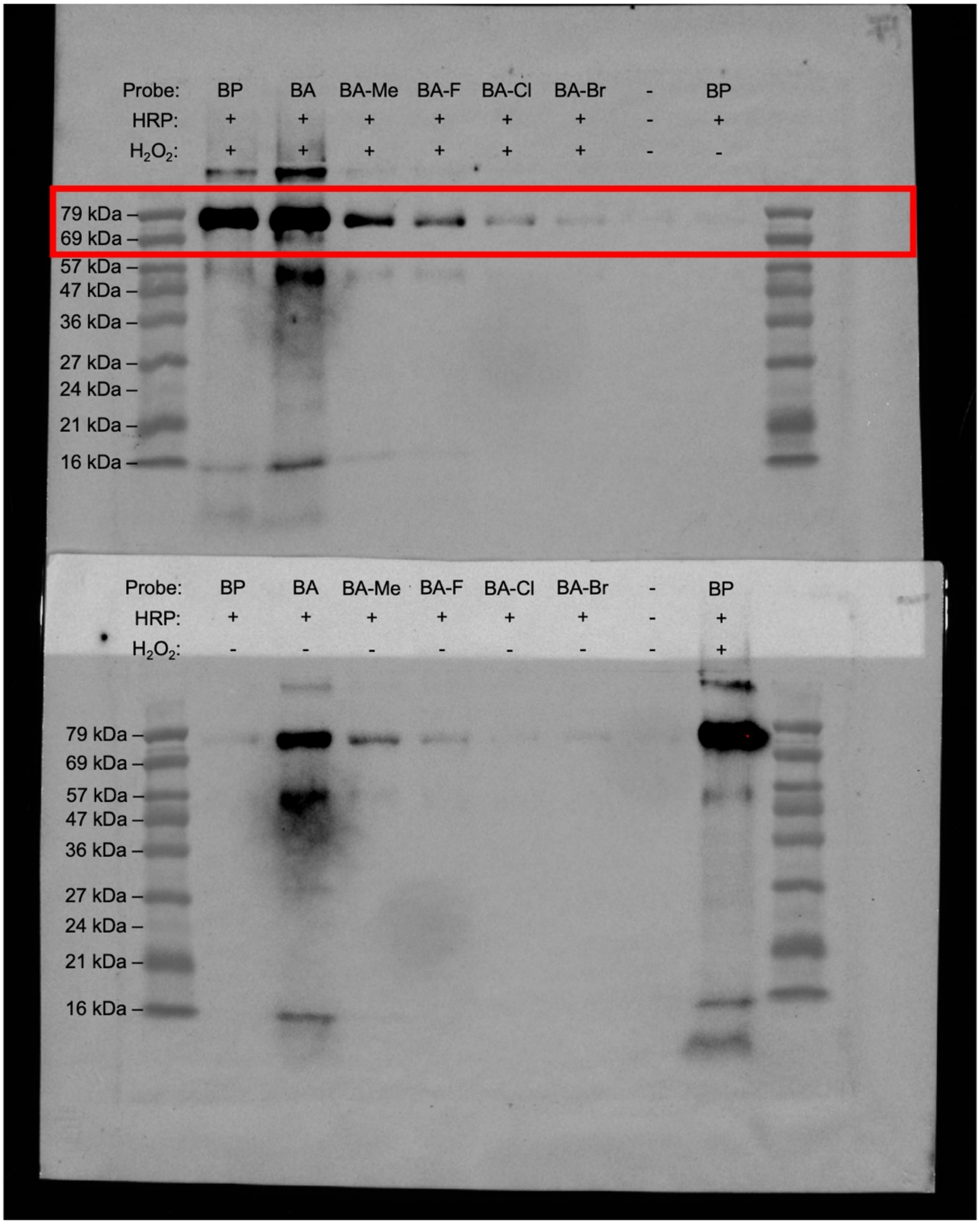

Figure S32. Raw western blot image for Figure 3a. Blot shows the greyscale blot for labelled BSA via HRP-mediated labelling reaction. Upper blot, Lane 1: Protein ladder, Lane 2-7: BSA labelled by horseradish peroxidase-mediated labelling reaction with BP, BA, BA-Me, BA-F, BA-Cl, and BA-Br respectively, Lane 8: Standard BSA, Lane 9: Negative control for BP-labelling BSA condition, Lane 10: Protein ladder. Lower blot, Lane 1: Protein ladder, Lane 2-7: Negative control for BSA labelling conditions by BP, BA, BA-Me, BA-F, BA-Cl, and BA-Br respectively, Lane 8: Standard BSA, Lane 9: BP-labelled BSA by HRP-mediated labelling reaction, Lane 10: Protein ladder.

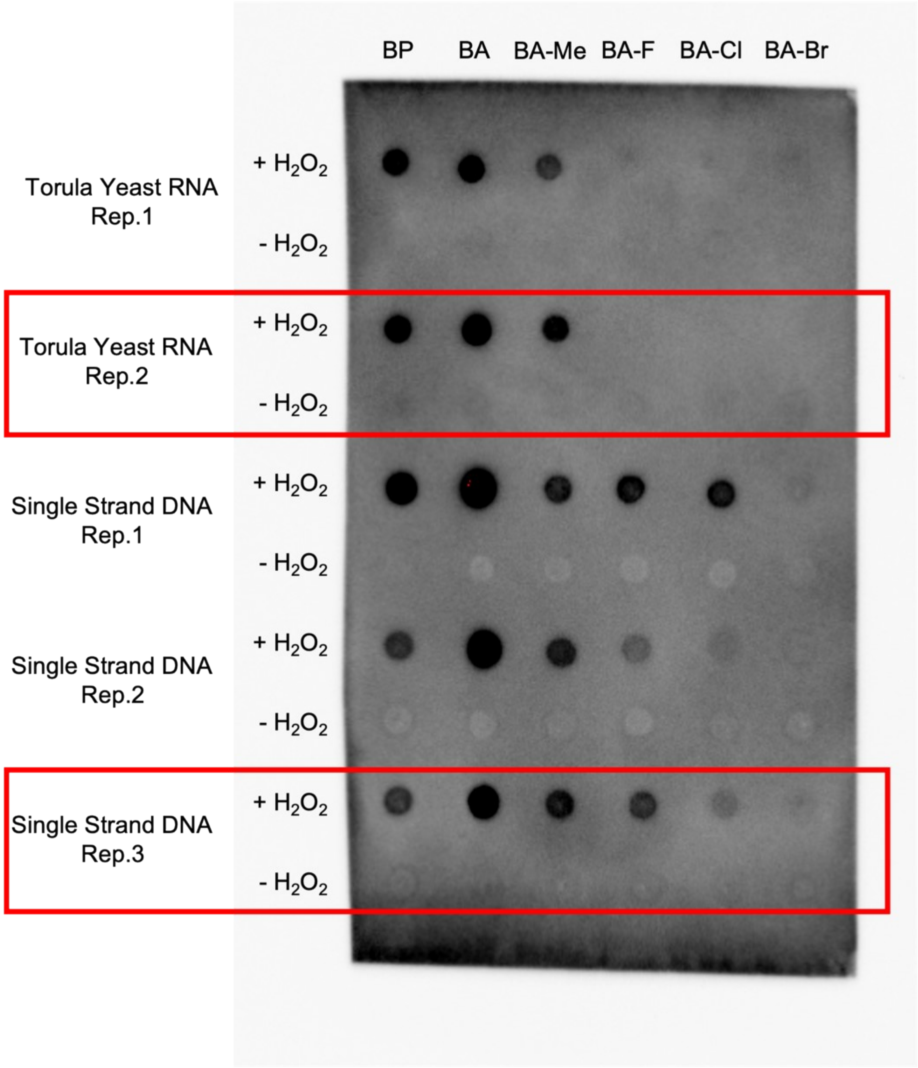

Figure S33. Raw dot blot image for Figure 3b and 3c. Row 1: Treated conditions for Torula Yeast RNA (rep. 1), Row 2: Untreated conditions for Torula Yeast RNA (rep. 1), Row 3: Treated conditions for Torula Yeast RNA (rep. 2), Row 4: Untreated conditions for Torula Yeast RNA (rep. 2), Row 5: Treated condition for Single Strand DNA (rep. 1), Row 6: Untreated condition for single strand DNA (rep. 1), Row 7: Treated conditions for single strand DNA (rep. 2), Row 8: Untreated conditions for single strand DNA (rep. 2), Row 9: Treated condition for single strand DNA (rep. 3), Row 10: Untreated condition for single strand DNA (rep. 3), From left to right: BP, BA, BA-Me, BA-F, BA-Cl, and BA-Br.

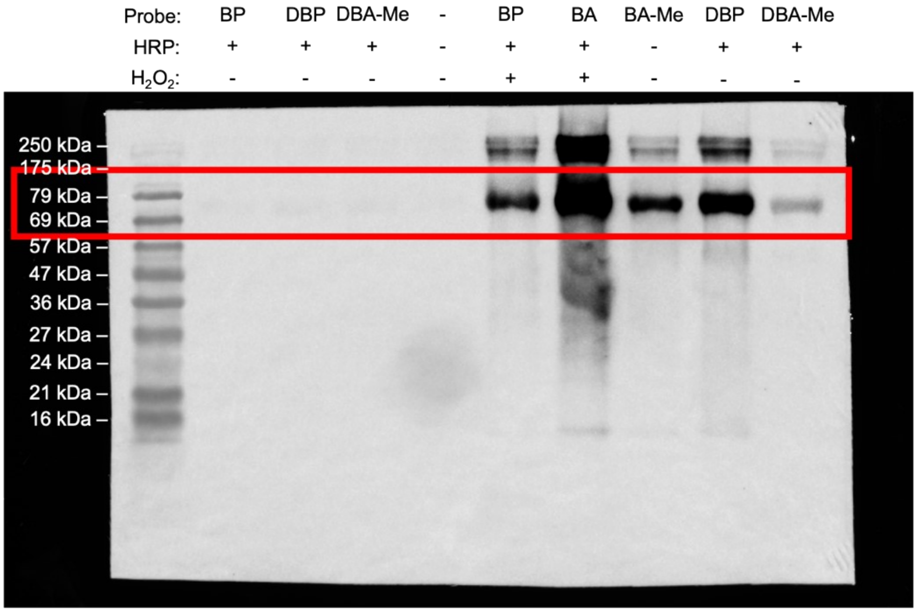

Figure S34. Raw western blot image for Figure 5b. The blot in the greyscale blot for labelled BSA via HRP-mediated labelling reaction was stacked with the chemiluminescence image. Lane 1: Protein ladder, Lane 2-4: probe-labelled BSA without H_2O_2. Lane 5: standard BSA, Lane 6-10: probe-labelled BSA with BP, BA, BA-Me, DBP, and DBA-Me, respectively, in the presence of H_2O_2.

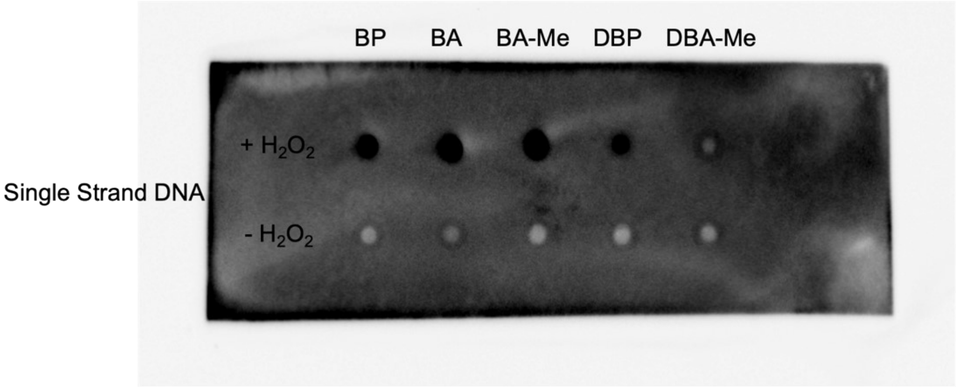

Figure S35. Raw dot blot image for Figure 5c. Row 1: Treated conditions for single strand DNA, Row 2: Untreated conditions for single strand DNA, From left to right: BP, BA, BA-Me, DBP, and DBA-Me.

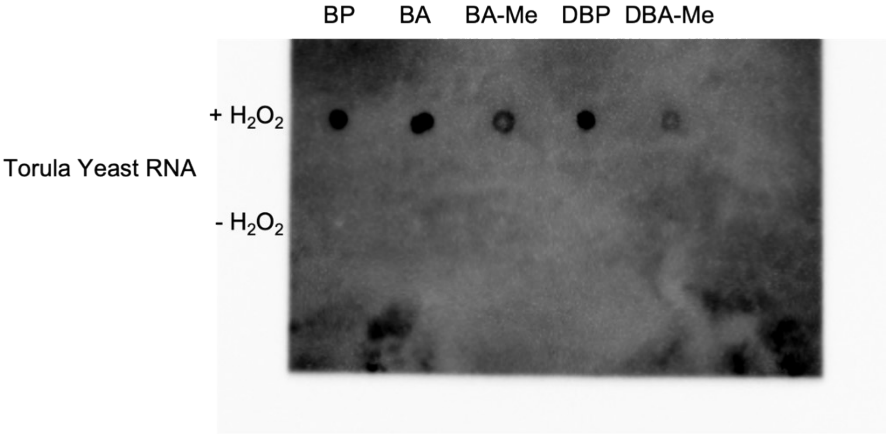

Figure S36. Raw dot blot image for Figure 5d. Row 1: Treated condition for Torula Yeast RNA, Row 2: Untreated condition for Torula Yeast RNA, From left to right: BP, BA, BA-Me, DBP, and DBA-Me.

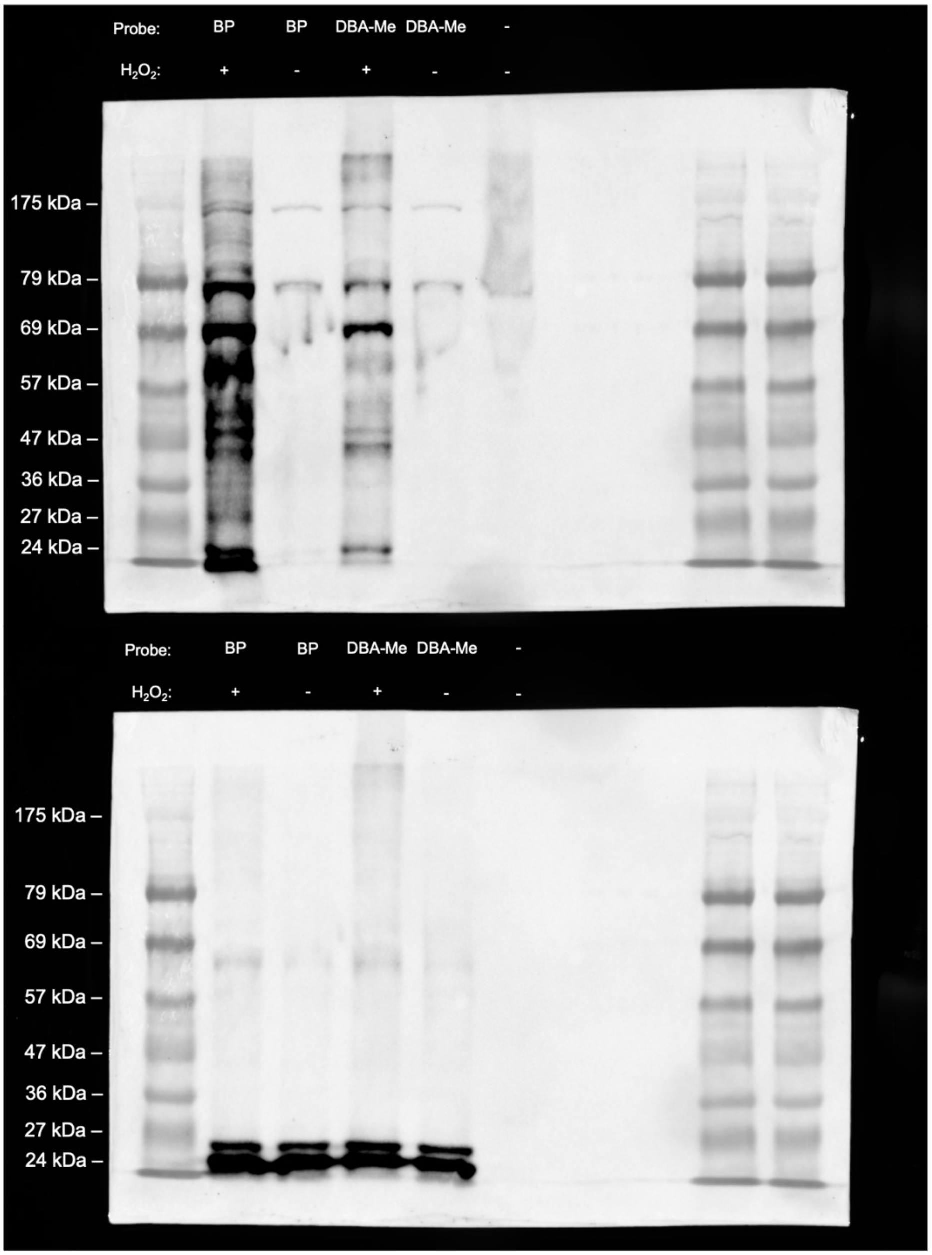

Figure S37. Raw western blot image for APEX2-mediated mitochondrial matrix protein labelling in HEK293FT in Figure 7. SA-HRP blot shown in the greyscale blot was stacked with the brightfield image. Upper panel shows SA-HRP staining while lower panel displays anti-V5 staining. Lane 1: Protein ladder, Lane 2: Treated condition of BP, Lane 3: Untreated condition of BP, Lane 4: Treated condition of DBA-Me, Lane 5: Untreated condition of DBA-Me, Lane 6: Cell lysate of Mito-V5-APEX2-expressing HEK293FT, Lane 7-8: Blank, Lane 9-10: Protein ladder.

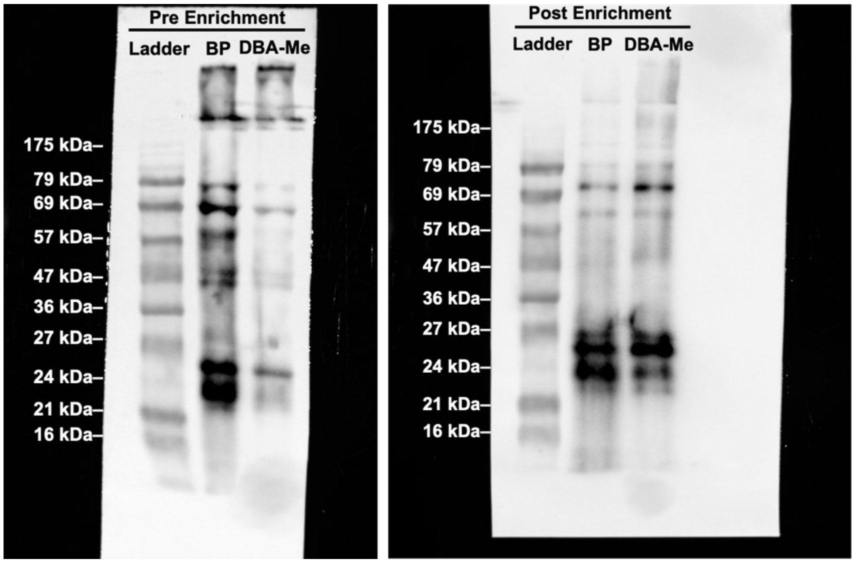

Figure S38. Raw western blot image for APEX2-mediated mitochondrial matrix protein labelling in HEK293FT in Figure 9. Western blot image for pre (left)- and post (right)-streptavidin enrichment of APEX2-mediated mitochondrial matrix protein labelling in HEK293FT. SA-HRP images were stacked with the brightfield image. Blot shows SA-HRP staining. Lane 1: Protein ladder, Lane 2: **BP**, Lane 3: **DBA-Me**.

Additional References for supplementary material

1. G. Nickerl, A. Notzon, M. Heitbaum, I. Senkovska, F. Glorius and S. Kaskel, *Crystal Growth & Design*, 2013, **13**, 198-203.
2. M. Cigl, A. Bubnov, M. Kašpar, F. Hampl, V. Hamplová, O. Pacherová and J. Svoboda, *Journal of Materials Chemistry C*, 2016, **4**, 5326-5333.
3. V. Pappula and S. Adimurthy, *RSC Advances*, 2016, **6**, 90184-90187.
4. Y. Zhou, G. Wang, P. Wang, Z. Li, T. Yue, J. Wang and P. Zou, *Angewandte Chemie International Edition*, 2019, **58**, 11763-11767.
5. S.-Y. Lee, M.-G. Kang, S. Shin, C. Kwak, T. Kwon, J. K. Seo, J.-S. Kim and H.-W. Rhee, *Journal of the American Chemical Society*, 2017, **139**, 3651-3662.
6. S. Krobthong, Y. Yingchutrakul, P. Wongtrakoongate, H. Chuntakaruk, T. Rungrotmongkol, C. Chaichana, T. Mahatnirunkul, T. Chomtong, K. Choowongkomon and C. Aonbangkhen, *Foods*, 2023, **12**, 780.
7. S. Krobthong, Y. Yingchutrakul, C. Butkinaree, C. Tantapakul, P. Moung-ngam, N. Boonhaijaroen, C. Aonbangkhen, S. Channumsin and T. Tulyananda, *Acta Astronautica*, 2025,
